# A deep learning-based prognostic approach for predicting turbofan engine degradation and remaining useful life

**DOI:** 10.1038/s41598-025-09155-z

**Published:** 2025-07-19

**Authors:** Samiha M. Elsherif, Bassel Hafiz, M. A. Makhlouf, Osama Farouk

**Affiliations:** Information Systems Department, Faculty of Computers and Informatics, Suez Canal University, Ismailia, 41522 Egypt

**Keywords:** C-MAPSS, Remaining useful life prediction, CNN, LSTM, Prognostics, Computer science, Information technology

## Abstract

Predicting the Remaining Useful Life (RUL) of turbofan engines can prevent air disasters caused by component degradation. It is an important procedure in prognostics and health management (PHM). Therefore, a deep learning-based RUL prediction approach is proposed. The CMAPSS benchmark dataset is used to determine the RUL of aviation engines, focusing specifically on the FD001 and FD003 sub-datasets.In this study, we propose a CAELSTM (Convolutional Autoencoder and Attention-based LSTM) hybrid model for RUL prediction. First, the sub-datasets are preprocessed, and a piecewise linear degradation model is applied. The proposed model utilizes an autoencoder followed by an LSTM layer with an attention mechanism, which focuses on the most relevant components of the sequences. A fully connected layer of the convolutional neural network is used to further process the important features. Finally, the proposed model is evaluated and compared with other approaches. The results show that the model surpasses state-of-the-art methods, achieving RMSE values of 14.44 and 13.40 for FD001 and FD003, respectively. Other evaluation criteria, such as MAE and scoring, were also used, with MAE achieving values of 10.49 and 10.68 for FD001 and FD003, respectively. The scoring achieved values of 282.38 and 264.47 for the same sub-datasets. These results highlight the model’s promise for improving prognostics and health management (PHM) systems, offering a dependable tool for predictive maintenance in aerospace and related fields. They also demonstrate the effectiveness and superiority of the model in enhancing aviation safety.

## Introduction

The fourth phase of the Industrial Revolution is being driven by the Industrial Internet of Things (also referred to as Industry 4.0)^1^. It will accelerate the transition of industries to a higher level and reshape economies, ushering in a new era of prosperity and competitiveness. A fundamental goal of Industry 4.0 is to increase asset uptime to improve productivity and decrease production and maintenance costs. Artificial intelligence (AI), the Industrial Internet of Things (IIoT), and cyber-physical systems (CPS) are recent technologies that have hastened the development of data-oriented applications like predictive maintenance (PdM). Maintenance is a top priority for companies that manage industrial resources, as effective maintenance predictions can help avoid critical failures and further losses.

The growing emphasis on the reliability and maintenance of complex systems like turbofan engines requires intelligent and autonomous approaches to managing the health of these safety-critical systems^2^. One approach is to use autonomous anomaly detection to monitor the health of turbofan engines. Early detection of anomalies in turbofan engines allows operators to take corrective actions and avoid catastrophic failures^2^. Prognostics and Health Management (PHM) has garnered significant attention in recent years, with RUL prediction being critical in PHM technology. Predicting the RUL of systems or components using sensor data or modeling can enhance maintenance practices and reduce downtime for aero-engines^3^. Prognostics is a field in aerospace that deals with predicting the future health and performance of important aircraft components or systems. It involves forecasting the remaining useful life (RUL) of components or systems, detecting impending defects or failures before they occur, and making informed decisions about maintenance, repair, and replacement plans. The goal is to ensure that aircraft operations are safe, reliable, and efficient while minimizing downtime and maintenance costs^4^. This prognosis is based on historical data, real-time monitoring, and an evaluation of the equipment’s condition and performance. Prognostics methodologies for calculating RUL fall into three categories: physical model-based, data-driven, and hybrid^5^.

The model-based technique involves analyzing the machine’s physical architecture and utilizing physics to create a mathematical model for RUL estimation. Mathematical models often make simplistic assumptions about handling uncertainty in complicated industrial machinery, which can severely limit these methodologies and reduce the accuracy of RUL forecasts^6^. Data-driven prognosis techniques use a variety of statistical and machine learning (ML) algorithms to identify trends or patterns in the base sensor data and predict the system’s RUL. These procedures are suitable for sophisticated industrial machinery and do not require a detailed understanding of the entire engine or the process^7^. The hybrid technique combines both data-driven and physics-based approaches^8^.

Many researchers have taken advantage of data-driven prognostic approaches over the last decade. These models analyze sensor data to determine the remaining useful life based on degradation trends and target trajectories. Deep learning techniques, such as autoencoders, Convolutional Neural Networks (CNNs), and Long Short-Term Memory (LSTM) networks, have shown effectiveness in computer vision, speech recognition, predictive maintenance, and other fields^9^. However, deep learning techniques require extensive offline training data, making it difficult to collect runtime-to-failure sensor data for new equipment. One option is to purposely run a new system until failure occurs, but this is a time-consuming, undesirable, and costly strategy. Due to these restrictions, researchers often prefer to use public databases for review. In this work, we employed the NASA C-MAPSS (Commercial Modular Aero Propulsion System Simulation) dataset, a tool for simulating turbofan engines, to generate the required data^4^. The details of this dataset are explained in section 4.1.

Several studies have been conducted to estimate the RUL of turbofan engines utilizing deep learning approaches such as CNNs, LSTMs, and their combinations and variants. LSTM networks have outperformed CNN-based models and have demonstrated outstanding results because they are well-suited for time-series data, can learn temporal features in multivariate systems, and minimize root mean square error with respect to target predictions^5^. While LSTMs are effective at capturing long-term dependencies, they suffer from vanishing gradients when processing long sequences, making it difficult to understand complex degradation patterns over extended operating cycles. Furthermore, LSTMs often have high computational costs and require significant memory, which makes them inefficient for real-time predictive maintenance applications. CNNs excel at extracting spatial and local properties from time-series data but struggle to model long-range dependencies. Their reliance on fixed-size receptive fields may limit their ability to detect changing degradation trends over time.

This paper proposes a combination of a convolutional autoencoder with an attention-based LSTM network for the RUL prediction of turbofan engines. Using a convolutional autoencoder in the context of time series data provides several benefits for various deep learning applications. It can learn hierarchical features from time series data at varying degrees of abstraction^10^. The convolutional layers capture patterns at different temporal scales, allowing the model to extract key information more efficiently. They are particularly useful for capturing temporal patterns in time series data and can learn to recognize patterns that repeat at specific time intervals, making them ideal for analyzing sequential data such as time series^11^.

Convolutional autoencoders minimize the dimensionality of time series data while retaining key temporal information. This helps in extracting important features and reducing noise in the data, which is essential for accurate predictions. They generalize well to previously unseen time series data, enabling excellent predictions even on fresh and unknown sequences. This capability is critical for developing reliable predictive maintenance models that can adapt to changing conditions^12^. Furthermore, the convolutional processes in autoencoders enable parallel processing, making them computationally efficient for analyzing time series data, resulting in faster training and inference times^13^. Convolutional autoencoders can act as an early warning system by detecting patterns that indicate potential failures or maintenance needs, thereby preventing costly breakdowns and ensuring optimal equipment operation^14^.

The Long Short-Term Memory (LSTM) network can learn the relationship between target RUL values and sensor data, but it often struggles to achieve optimal performance due to various limitations. These constraints can impair the functionality of an LSTM network. In this study, we focus on performing various preprocessing procedures on sensor data before it is fed into the model network. The proposed model, when paired with appropriate preprocessing techniques, has the ability to estimate the remaining useful life with high accuracy, with full details provided in “Data preprocessing”. A modified piecewise linear degradation model is introduced to determine the beginning point of degradation. It has been demonstrated that the starting point of degradation, also known as the initial RUL, significantly impacts the accuracy of RUL predictions. This improved degradation model quickly calculates the starting point of degradation, resulting in accurate RUL predictions when combined with additional preprocessing techniques and the model network.

Our key contributions in this study are listed as follows:

1. A piecewise linear degradation model is proposed.
2. A model network with effective prepossessing steps are proposed which composed of several essential components:
  - The encoder part of the model consists of convolutional layers followed by max-pooling layers, which help extract hierarchical features from the input time series data. The decoder part then reconstructs the input data from the encoded representation, enabling feature extraction and noise reduction.
  - After the autoencoder, an LSTM layer is utilized to process the encoded output, capturing temporal dependencies and patterns in the data, which is crucial for time series analysis.
  - An attention mechanism is employed to focus on important parts of the LSTM output, enhancing the model’s ability to weigh and select relevant information, aiding in capturing long-term dependencies effectively.
  - Following the attention mechanism, fully connected layers are used to further process the extracted features and generate a final prediction. The dropout layers help prevent overfitting during training.
  - The model is compiled using the Adam optimizer with specified learning rate and Mean Squared Error loss function. Additionally, Mean Absolute Error and Root Mean Squared Error metrics are tracked during training to evaluate model performance.
3. Hyper-parameters for the proposed prediction model were determined through multiple experiments. The experimental results indicate that the Model greatly improves remaining useful life prediction performance.

The proposed model is a considerable improvement over typical LSTM and CNN architectures due to its novel blend of techniques that capitalize on the strengths of each approach. Here are a few crucial points that demonstrate why CAELSTM is superior:

1. The CAELSTM employs convolutional layers to effectively capture local characteristics and patterns in input data. This functionality is especially useful for sequential data, such as time series, in which local correlations play an important role. Traditional LSTM models, which rely mostly on internal-state mechanisms, may miss these essential local patterns.
2. CAELSTM may learn hierarchical features at different levels of abstraction by stacking multiple convolutional layers, which improves the model’s capacity to interpret complex data structures. Traditional LSTMs lack this hierarchical feature learning capability, limiting their effectiveness on complicated data sets.
3. Incorporating pooling layers into the CAELSTM architecture allows for effective dimensionality reduction, which reduces computational complexity while keeping key features. In contrast, typical LSTMs do not contain such methods by default, potentially leading to more complex and noisy inputs.
4. Combining the Encoder-Decoder Framework increases the model’s resistance to noise and aids in collecting important representations of the input data, resulting in enhanced overall performance.
5. The addition of an attention mechanism enables CAELSTM to concentrate on the most important parts of the input sequence, improving the model’s predicted accuracy. This selective emphasis mitigates the influence of irrelevant data, which is a major issue in traditional LSTM models that analyse full sequences evenly.
6. CAELSTM’s hybrid nature allows it to adapt to a wide range of sequential data tasks, including time series forecasting and natural language processing. It combines the benefits of CNNs for feature extraction with LSTMs for sequence modeling to create a flexible framework that can be customized for unique purposes.

CAELSTM combines the strengths of convolutional networks and LSTMs, enhanced by the use of an attention mechanism, resulting in a model that not only excels in capturing complex relationships in sequential data, but also improves upon the limitations of traditional LSTMs and CNNs. Its architecture allows for effective feature extraction, contextual understanding, and robustness, making it an advanced choice for a wide range of applications.

Both CAELSTM and transformer-based models provide distinct advantages for time series analysis, catering to a variety of demands and situations. CAELSTM is especially useful when smaller datasets are available and interpretability is critical, whereas transformer models excel at handling huge datasets with comprehensive feature representations, but may necessitate additional processing resources. Exploring the strengths of both paradigms may reveal new opportunities for future research and model development. Vision Transformers (ViTs) use the transformer design that was originally developed for natural language processing. They use the self-attention mechanism to process image data, using image patches as tokens. The ideas of ViTs can be applied to time series data. BERT is a transformer-based model specifically built for NLP tasks. It makes use of a bidirectional attention mechanism, allowing the model to learn a word’s context from both the left and right context. Bidirectional Encoder Representations from Transformers (BERT) can also be used for time series analysis by following the same contextualised learning principles. Both Vision Transformers (ViTs) and BERT have different advantages in predictive maintenance, but they are not without limits. ViTs often require a considerable quantity of data to train adequately, which may be a limitation in some predictive maintenance scenarios. Leveraging ViTs may necessitate more complex hyperparameter tweaking. BERT can be computationally demanding in terms of memory and computing, necessitating large resources for training and inference. Achieving optimal performance may need careful tuning and experimentation with different architectures and hyper-parameters^15–17^.

The rest of this paper is organized as follows: the literature review is presented in “Literature review”. Next, the proposed methodology is detailed in “Proposed methodology”, followed by the experimental verification in “Experimental verification”. Then, the ablation study results for the model without attention are discussed in “Ablation study results of CAELSTM Model without attention”. Finally, the conclusion is provided in “Conclusion”.

## Literature review

In this section, we review research studies that have utilized popular deep learning algorithms to predict the remaining useful life (RUL). Most of these studies have employed the C-MAPSS dataset.

In^18^, researchers proposed a deep CNN-based regressor for estimating the RUL of complex systems using multivariate time series data. The proposed deep architecture leverages convolution and pooling layers to identify patterns in sensor signals across different time scales. These detected patterns are then aggregated and used in the RUL estimation model. The model’s performance was evaluated using Root Mean Square Error (RMSE) and a scoring function. The obtained RMSE values were 18.45 and 19.82, while the S-scores were 1299 and 1600 for the FD001 and FD003 datasets, respectively.

The study in^19^ introduced MODBNE, a multi-objective evolutionary ensemble learning method that simultaneously evolves multiple Deep Belief Networks (DBNs) while balancing accuracy and diversity as competing objectives. The DBNs are combined to form an ensemble model for RUL estimation, with weights optimized using a single-objective Differential Evolution (DE) algorithm that minimizes average training error. The model’s performance was assessed using RMSE and a scoring function, yielding RMSE values of 15.04 and 12.51, and S-scores of 334 and 422 for FD001 and FD003, respectively. In future research, the authors plan to extend their approach to classify various predictive health conditions. They also propose enhancing MODBNE’s multi-objective optimization into a many-objective optimization framework, considering additional valuable yet competing objectives beyond accuracy and diversity. Additionally, GPU-based implementations of MODBNE will be explored to improve computational efficiency.

A deep learning approach for RUL estimation using sequence information was presented in^20^. The proposed model consists of LSTM layers followed by feedforward networks. The model’s performance was evaluated using RMSE and a scoring function, with obtained RMSE values of 16.14 and 16.18, and S-scores of 338 and 852 for FD001 and FD003, respectively.

Researchers in^21^ proposed a deep learning strategy using an RNN with two LSTM layers to address the remaining useful life (RUL) estimation problem for aircraft engines. The LSTM structure effectively captures relationships between data points across time. The proposed model was evaluated using RMSE and a scoring function, achieving an RMSE of 19.64 and a score of 838.99 for the FD001 dataset.

The study in^22^ introduced a hybrid deep LSTM technique for machinery prognosis. This approach combines LSTM layers with traditional neural networks to extract meaningful information from sequential sensory data. Additionally, the proposed technique performs sensor fusion by integrating data from multiple sensors at the data level, which enhances prognostic performance. The model is trained directly on raw sensory data for RUL prediction.

An ensemble recurrent neural network-based approach for RUL prediction in aircraft engines was proposed in^23^. The model utilizes preprocessed and selected sensor data as input. Three types of RNN models with different structures—LSTM, GRU, and ordinary RNN neurons—were developed. The sensor data from the initial stage was used to train these three RNN models, which were then combined using ensemble learning with a stacking architecture. The stacking framework employed a random forest algorithm as its second-level meta-learner. The ensemble model integrates features extracted by the three recurrent neural networks. The model’s performance was evaluated using RMSE, yielding results of 14.57 and 14.92 for FD001 and FD003, respectively.

Researchers in^24^ proposed a bidirectional LSTM-based autoencoder for RUL prediction. The model was evaluated using two publicly available run-to-failure datasets, with performance assessed through RMSE and a scoring function. The obtained results were an RMSE of 14.70 and a score of 273.00 for the FD001 dataset.

An attention-based deep learning framework for RUL prediction was introduced in^25^. Long Short-Term Memory (LSTM) networks were employed to extract sequential features from raw sensory data. The proposed attention mechanism prioritized significant features and time steps by assigning higher weights to them. Extensive experiments on two real-world datasets (FD001 and FD004) demonstrated that the proposed technique outperformed previously mentioned methods in the study. The model was evaluated using RMSE and a scoring function, achieving an RMSE of 14.53 and a score of 322.44 for FD001.

The study in^26^ proposed the use of a Time Series Chain Graph (TSCG) to effectively detect potential degradation scenarios by modeling dependencies between time-varying risk factors and performance data. The study outlines the process of building a model based on observed time-series data and applying it to RUL prediction. The inherent complexity of the TSCG structure enhances its ability to distinguish between different degradation conditions, making RUL estimation more reliable. The model was evaluated using RMSE and a scoring function, achieving an RMSE of 17.44 and a score of 468.49 for FD001.

Scholars in^2^ proposed a semi-supervised autoencoder-based technique for anomaly detection in turbofan engines. The study applied an autoencoder with Bayesian hyperparameter tuning to enhance model performance and examined the impact of removing redundant features. Two approaches were evaluated: one retaining all features and another removing redundant ones. Results indicated that the autoencoder model performed optimally without feature removal. Pearson’s correlation was used to analyze the effect of removing redundant features on model performance. The study utilized a Tree-Structured Parzen Estimator (TPE) to create a surrogate model of the target function, leveraging Bayes’ rule. The model was evaluated using F1-score, precision, and recall. The optimized autoencoder demonstrated effectiveness in real-world anomaly detection, achieving an F1-score of 0.892, precision of 0.896, and recall of 0.724 for the model without feature removal.

In^27^, researchers proposed an LSTM-AE-based framework for precise and reliable anomaly detection in complex aircraft systems. The model employs an Autoencoder (AE) and Long Short-Term Memory (LSTM) network. The dataset used in this study was collected from four single-aisle twin-engine commercial aircraft, comprising approximately 5,800 flights over one year. The results indicate that the calculated Health Index (HI) effectively characterizes the aviation system’s health state, allowing for high-confidence diagnosis of distinct fault types. The study suggests that further research is needed to establish a warning threshold by balancing false alarm costs and safety margins. This model is applied using Aircraft Condition Monitoring System (ACMS) parameters linked to the air bleed system.

Researchers in^28^ proposed a hybrid model based on LSTM-RNN-HMM for estimating the remaining life of aviation engines. The approach integrates a Hidden Markov Model (HMM), a Recurrent Neural Network (RNN), and an LSTM network. This research compares the performance of HMM and LSTM models, highlighting similarities in their hidden state structures. The hybrid HMM-LSTM strategy combines the interpretability of HMM with the strong predictive capabilities of LSTM. The study uses the C-MAPSS dataset and evaluates the model using Remaining Useful Life error (RULerror), Mean Square Error (MSE), and F1-measure. The obtained F1-measure is 0.781. The researchers suggest that, in certain cases, a smaller hybrid model may be effective. They implemented their model using the hmmlearn package in Python and TensorFlow.

In^29^, a predictive maintenance strategy named DAE-LSTMQR-KDE was proposed. The study also introduced a replacement cost function and an ordering cost function. The model utilizes a Deep Autoencoder (DAE), an LSTM network, deep learning-based quantile regression, and Kernel Density Estimation (KDE). The C-MAPSS dataset was used for evaluation. Results indicate that the proposed prediction and maintenance approach is both promising and competitive in terms of improving prediction accuracy and reducing maintenance costs. The suggested predictive maintenance strategy can effectively adapt to cost fluctuations. The model was evaluated using failure prediction accuracy, root mean square error (RMSE), score function, coverage probability, mean width, and coverage width-based criteria. The implementation was carried out using MATLAB’s Deep Learning Toolbox. The obtained results for RMSE and S-Score are 13.58 and 246.59, respectively, for the FD001 dataset.

Researchers in^30^ proposed a PCA-LSTM method for predicting the RUL of aero-engines. The approach analyzes multi-sensor monitoring data using Principal Component Analysis (PCA) to reduce data dimensionality and improve prediction performance by extracting correlations between engine sensor data. The model integrates an LSTM network with PCA. The C-MAPSS dataset was used for training. The proposed method enhances training speed, accuracy, and prediction stability. It was compared to various algorithms, including Support Vector Machines (SVM) and LSTM. RMSE and a computed score function were used to evaluate performance. The obtained RMSE and S-Score are 19.43 and 674.79, respectively, for the FD001 dataset.

A hybrid deep neural network model was introduced in^3^ to enhance prognosis accuracy for equipment RUL prediction. The model employs a Convolutional Neural Network (CNN) to extract local features and a Bidirectional Gated Recurrent Unit (BGRU) to capture both forward and backward long-term dependencies. Additionally, a Self-Attention (SA) mechanism is applied to assign appropriate weights to different features. This approach improves performance and generalization for high-dimensional data by eliminating redundant information, reducing computation time, and extracting both spatial and temporal features from monitoring inputs. By accurately predicting engine RUL, the model contributes to improved maintenance strategies and operational efficiency, reducing economic losses and aviation equipment failures. Performance was evaluated using the Mean Squared Error (MSE) function on the C-MAPSS dataset. The obtained RMSE and S-Score are 13.88 and 248 for FD001, and 14.85 and 295 for FD003.

A concurrent semi-supervised framework for RUL prediction was proposed in^31^. The framework consists of two components: an unsupervised component using a Variational Autoencoder (VAE) in the encoder layers and a supervised component incorporating CNNs, LSTMs, and dense layers. The proposed technique outperforms existing baseline models, exhibiting greater stability and accuracy compared to related approaches. The study used the C-MAPSS dataset, and model performance was evaluated using the NASA score function. The obtained RMSE and S-Score are 12.19 and 208.11 for FD001, and 12.92 and 245.45 for FD003. Given the large amount of data stored for each engine, the researchers recommend that future studies explore modeling based on forecast values across all time series in the training set to maximize data utilization.

Researchers in^5^ proposed a model that combines effective pre-processing techniques with a Long Short-Term Memory (LSTM) network. They introduced an improved piecewise linear degradation model to determine the initial point of deterioration and assign RUL target labels. The C-MAPSS dataset was used for evaluation. The study demonstrates that integrating an LSTM model with appropriate pre-processing techniques significantly enhances RUL prediction accuracy. Root Mean Square Error (RMSE) and a scoring function were used to evaluate the model’s performance. The experiments were conducted using MATLAB. The obtained RMSE values were 7.78 and 8.03, while the S-Scores were 100 and 104 for datasets FD001 and FD003, respectively. Since prognostics often lacks real-world data for industrial machines, especially in malfunctioning conditions, the study highlights the need for novel approaches that can effectively train on small datasets while accurately predicting outcomes on large-scale practical data.

A model based on variational encoding and a novel method for regularizing latent representations was proposed in^32^. RMSE and a scoring function were used to assess the model’s performance. The obtained RMSE values were 15.81 and 14.88, while the S-Scores were 326 and 722 for datasets FD001 and FD003, respectively.

Researchers in^33^ proposed an attention-based Gated Recurrent Unit (ABGRU) algorithm to predict the remaining useful life (RUL) of aero-engines. The approach integrates attention-based seq2seq modeling and GRU-based feature fusion with the piecewise linear degradation principle. The C-MAPSS dataset (FD001 and FD003) was used for evaluation. The results show that the proposed model outperforms state-of-the-art methods referenced in the literature. The performance was assessed using RMSE and a scoring function, yielding RMSE values of 12.83 and 13.23, with S-Scores of 221.54 and 279.18 for FD001 and FD003, respectively.

In^34^, researchers presented a dynamic predictive maintenance strategy for estimating RUL using a deep learning ensemble method. The approach employs a CNN and Bi-LSTM to predict RUL while incorporating order, stock, and maintenance decision-making. Experimental results on the NASA turbofan engine dataset demonstrate the strategy’s effectiveness compared to existing methods. The study underscores the importance of integrating system mission cycles into data-driven predictive maintenance techniques. The proposed method aims to minimize maintenance costs while enhancing system reliability. Future research will focus on refining RUL prediction algorithms and incorporating decision-maker preferences. The obtained RMSE for FD001 was 13.22, with an S-Score of 232.24.

A prediction model using CNN-LSTM-Attention was proposed in^35^. CNN was used to extract local features from aero-engine sensor data, while the LSTM-Attention network analyzed data characteristics to forecast engine life. Comparative studies were conducted to evaluate different RUL prediction models after ablation analysis. Experimental results demonstrated that the proposed model achieved accurate predictions across four datasets, with RMSE values of 15.977, 14.452, 13.907, and 16.637.

The study in^36^ highlights the importance of predictive maintenance in industrial applications and the role of machine learning (ML) methods in estimating RUL. A case study using NASA’s C-MAPSS dataset demonstrates the successful implementation of ML algorithms, including the use of Particle Swarm Optimization for parameter tuning. The study validates the effectiveness of ML techniques in RUL prediction, with Logistic Regression, Support Vector Machine, and Random Forest classifiers yielding promising results. The research focuses on the transition from reactive to proactive maintenance, ensuring improved machine reliability, durability, and efficiency.

Researchers in^37^ introduced a data-driven maintenance scheduling approach for aviation engines, incorporating deep learning to predict RUL. A deep learning ensemble model, combining CNN and Bi-LSTM-AM, was proposed to enhance RUL prediction accuracy. The study employed Bayesian optimization to fine-tune hyperparameters in the ensemble model. As an aircraft engine’s RUL decreases over time, a maintenance alarm threshold is triggered. Once this threshold is reached, maintenance scheduling is initiated. To optimize the scheduling process, a mixed-integer linear programming (MILP) model was developed to minimize total maintenance time. The results indicate that the proposed predictive maintenance scheduling framework effectively monitors aircraft engine health in real time, thereby reducing maintenance downtime.

## Proposed methodology

We propose a CAELSTM (Convolutional Autoencoder and Attention-Based LSTM) hybrid model for Remaining Useful Life (RUL) prediction. This model features an autoencoder-like structure, followed by an LSTM layer and an attention mechanism for effective sequence processing and prediction.

The architecture begins with convolutional layers for feature extraction, followed by max-pooling layers to downsample the data. The encoder compresses the input data into a latent representation, while the decoder reconstructs it to its original shape using upsampling layers. The encoded output is then passed to an LSTM layer, enabling the model to learn from sequential data. An attention mechanism focuses on the most relevant parts of the input sequence, calculating attention weights to generate a context vector that captures crucial information. This vector is processed through fully connected layers, culminating in a linear activation output layer that produces the final prediction.

In terms of computational efficiency, the CAELSTM model outperforms traditional LSTM and CNN architectures. By integrating convolutional and LSTM layers, it effectively extracts spatial features while capturing temporal dependencies. This hybrid approach leverages the strengths of both architectures: traditional LSTMs process data sequentially, often leading to longer training times, while CAELSTM’s convolutional layers extract essential features in parallel, improving overall efficiency. Although the model requires more memory due to an increased number of parameters, this trade-off is justified by its enhanced predictive accuracy. We monitored memory usage during training and found that CAELSTM demonstrated efficient resource management while delivering superior performance.

**Fig. 1:**
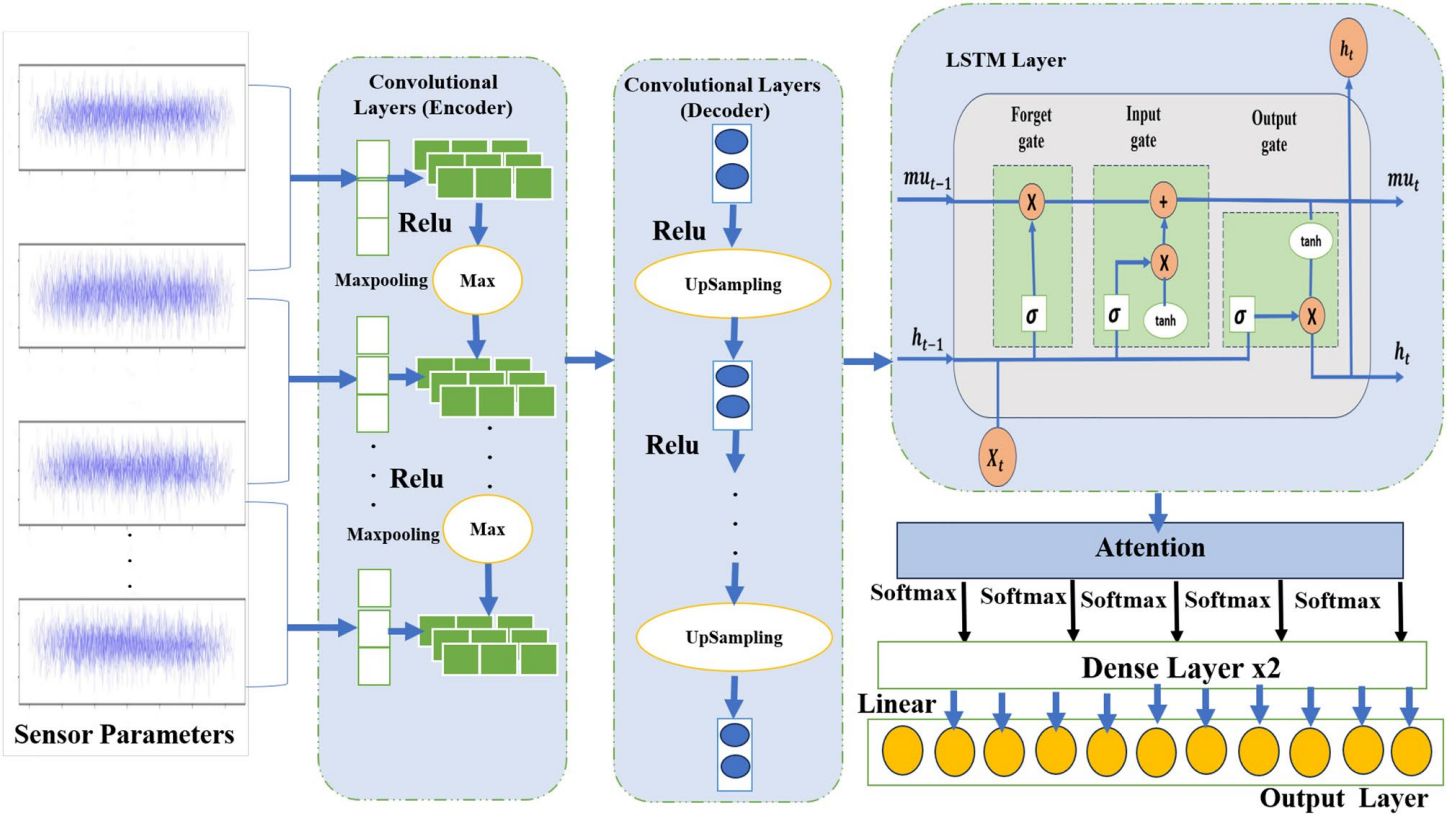
Proposed model.

To mathematically represent the proposed RUL prediction model, we break it down into key components: the convolutional autoencoder for feature extraction, LSTMs for sequence learning, an attention mechanism for feature weighting, and fully connected layers for processing. A detailed introduction to the proposed approach is provided in Table 1 and Figure 1. The CAELSTM model is summarized in Algorithm 1.

**Table 1:** The architecture of the proposed model.

	Layers	Parameters
Autoecoder encoder layers	Conv1D	Filters=128, kernal=3, Activation=ReLU, Padding=Same
	Conv1D	Filters=128, kernal=3, Activation=ReLU, Padding=Same
	Conv1D	Filters=256, kernal=3, Activation=ReLU, Padding=Same
	MaxPooling1D	Pool Size= 2, Padding= Same
Autoencoder decoder Layers	Conv1D	Filters=128, kernal=3, Activation=ReLU, Padding=Same
	Conv1D	Filters=128, kernal=3, Activation=ReLU, Padding=Same
	Conv1D	Filters=128, kernal=3, Activation=ReLU, Padding=Same
	UpSampling1D	Size=2
LSTM	LSTM	Units= 128, Return Sequences True
Attention Mechanism	TimeDistributed Layer	Dense Layer units=1, Activation=Tanh
	Flatten Layer	
	Activation Layer	Activation= Softmax
	RepeatVector Layer	128
	Permute Layer	[2, 1]
	Multiply Layer and Lambda Layer	
Fully Connected Layers	Dense Layer	Units= 200, Activation= ReLU
	Dropout Layer	Rate= 0.4 (FD001), Rate=0.6 (FD003)
	Dense Layer	Units= 100, Activation= ReLU
	Dropout Layer	Rate= 0.4 (FD001), Rate=0.6 (FD003)
Output Layer	Dense Layer	Units= 1, Activation= Linear
Final Model Compilation	Optimizer	Adam
	Learning Rate	0.001 (FD001) and 0.0001 (FD003)
	Loss Function	Mean Squared Error (MSE)
	Metrics	Mean Absolute Error, Root Mean Squared Error

**Algorithm 1:**
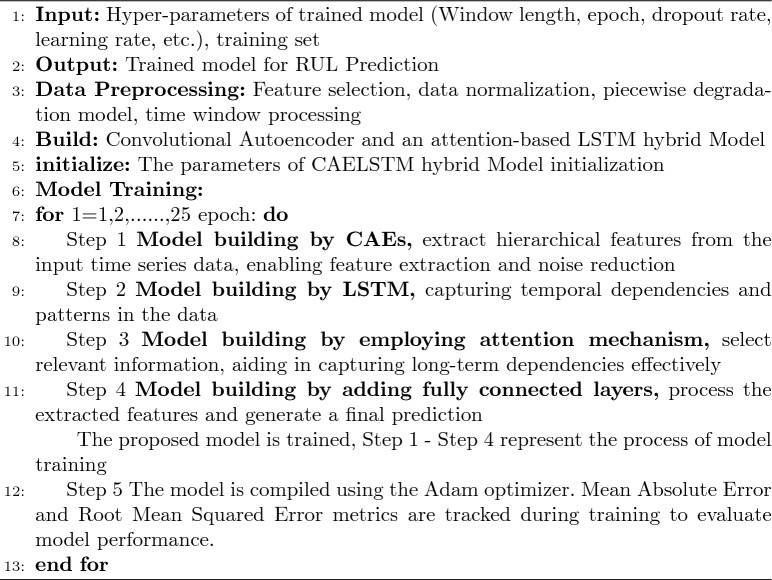
Proposed a CAELSTM hybrid model network.

1. Convolutional autoencoder part: the input data is passed through a series of convolutional layers to extract features. Let’s denote the input data as X and the output of the autoencoder as $\hat{X}$. The encoder part can be represented as follows^38^:
  1 $$\begin{aligned} h={f_{enc}(X)}=Conv(X) \end{aligned}$$
  The decoder part can be represented as:
  2 $$\begin{aligned} \hat{X}={f_{dec}(h)}=Deconv(h) \end{aligned}$$
2. LSTM layer: Let’s denote the output of the encoder as h. The LSTM layer processes this encoded data:
  3 $$\begin{aligned} LSTM_{out}=LSTM(h) \end{aligned}$$
3. Attention mechanism: Let’s denote the output of the LSTM layer as $LSTM_{out}$.Apply a dense layer with tanh activation
  4 $$\begin{aligned} A=Dense_{tanh}(LSTM_{out}) \end{aligned}$$
  Then, Apply softmax to get attention weights
  5 $$\begin{aligned} \alpha =Softmax(A) \end{aligned}$$
  After that, Apply attention to LSTM output
  6 $$\begin{aligned} Att=\sum _{i=1}^{T} \alpha \cdot LSTM_{outi} \end{aligned}$$
4. Fully Connected Layers: After attention mechanism, pass through fully connected layers
  7 $$\begin{aligned} FC_1=RELU(Dense_{200}(Att)) \end{aligned}$$
  8 $$\begin{aligned} FC_2=RELU(Dense_{100}(FC_1)) \end{aligned}$$
5. Output Layers: Finally, the output layer can be represented as:
  9 $$\begin{aligned} \hat{Y}=RELU(Dense_{Linear}(FC_2)) \end{aligned}$$

### Convolutional autoencoders

CAEs (convolutional autoencoders)^39^ combine the advantages of convolutional neural networks (CNNs) and autoencoder architectures, making them particularly useful for various tasks. They are especially effective for processing data with spatial or sequential structures, such as images, audio, and time series.

The encoder layers, also referred to as convolutional layers, perform convolution operations on input data to extract meaningful features. Typically, the encoder consists of a series of convolutional layers, each followed by a pooling layer. The convolutional layers apply filters to capture important patterns in the input data, while the pooling layers reduce the spatial dimensionality of the feature maps, helping to retain essential information while improving computational efficiency.

The decoder layers, often called deconvolution layers, perform transpose convolution operations to reconstruct the output data from the compressed latent representation. This process is essentially the inverse of the encoder’s function. The decoder is typically composed of a series of upsampling layers followed by convolutional layers. The upsampling layers increase the spatial dimensionality of the feature maps, while the convolutional layers use filters to restore the original input structure. This allows the network to learn the upsampling process directly from the data during training.

Transpose convolution can also learn to fill in missing data or generate new data based on previously learned patterns, making it valuable for various applications^40^.

For the convolution operation, let X represent the input data. The output of a convolutional layer can be computed as:

10 $$\begin{aligned} Z=f(W * X + b) \end{aligned}$$

where, Z is the output feature map. f is the activation function. W is the filter weights. b is the bias term. * is the convolution operation. After that pooling operation is applied. MaxPooling or AveragePooling is often applied to reduce spatial dimensions.

11 $$\begin{aligned} Y=Maxpooling(Z). \end{aligned}$$

Then the decoding phase is applied. The encoded features are reconstructed back to the input data shape. Upsampling is used to increase the spatial dimensions. The output of the upsampling layer:

12 $$\begin{aligned} U=Upsampling(Y). \end{aligned}$$

Finally, The transposed convolution operation is used to upsample the feature maps. The output of a transposed convolutional layer:

13 $$\begin{aligned} \hat{X}=f(\hat{W}* U + \hat{b}) \end{aligned}$$

where, $\hat{X}$ is the output of the decoding phase. $\hat{W}$ is the decoder weights. $\hat{b}$ is the decoder bias term.

### Long short term memory

**Fig. 2:**
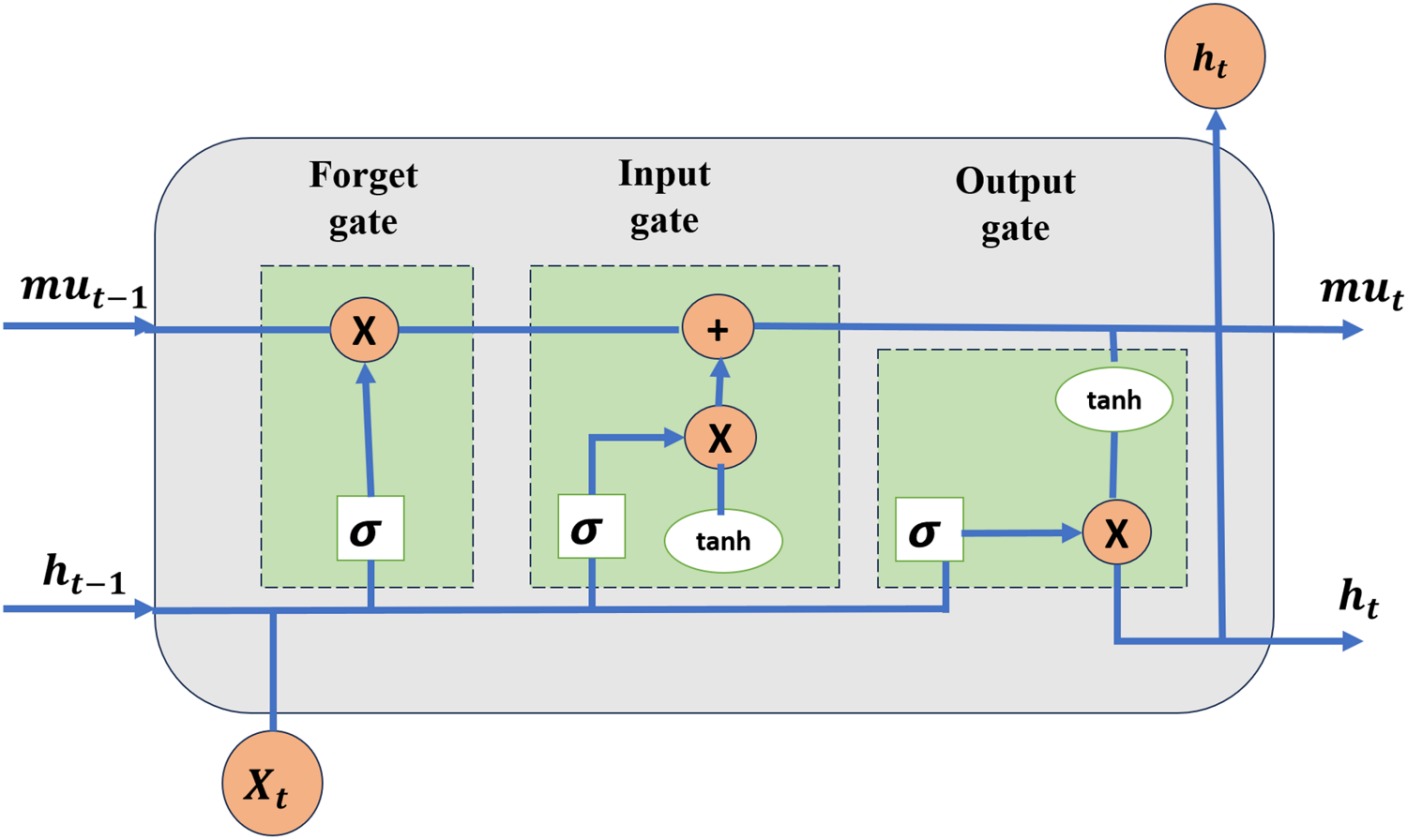
LSTM architecture (1 Layer with 128 units).

In 1997, Hochreiter and Schmidhuber introduced the Long Short-Term Memory (LSTM) network^41^, as shown in Fig. 2. LSTM is a widely used and reliable tool for predicting the remaining useful life (RUL) of systems. It employs a “gating” mechanism to regulate memory states during information transmission. This feature allows the network to selectively retain or discard information, enabling past data to be effectively transferred across time steps.

Compared to traditional deep learning methods, LSTM mitigates the vanishing gradient problem and enhances prediction accuracy. The LSTM architecture consists of three key components: the forget gate, input gate, and output gate. Their functions are as follows^42^:

1. The Forget gate ${fg_t}$ determines the quantity of information from the previous internal state $mu_{t-1}$to be forgotten.
2. The input gate ${ig_i}$ determines how much information can be saved from the current candidate state $mu_t$.
3. The output gate ${Og_t}$ controls how much information from the current internal state $mu_t$ must be communicated to the outside state $h_t$ .

The mathematical equations for the three gates are given below^42^:

14 $$\begin{aligned} {ig_i}= & \sigma ( {W_{ig}}{x_t}+{U_{ig}}{h_{t-1}}+{b_{ig}}) \end{aligned}$$

15 $$\begin{aligned} {fg_t}= & \sigma ( {W_{fg}}{x_t}+{U_{fg}}{h_{t-1}}+{b_{fg}}) \end{aligned}$$

16 $$\begin{aligned} {Og_t}= & \sigma ( {W_{og}}{x_t}+{U_{og}}{h_{t-1}}+{b_{og}}) \end{aligned}$$

17 $$\begin{aligned} {mu_t}= & fg_t \cdot mu_{t-1} + ig_t \cdot mu_t \end{aligned}$$

18 $$\begin{aligned} {h_t}= & Og_t \cdot tanh({mu_t}) \end{aligned}$$

19 $$\begin{aligned} {mu_t}= & tanh( {W_{mu}}{x_t}+{U_{mu}}{h_{t-1}}+{b_{mu}}) \end{aligned}$$

where, $x_t$ is the input variable. $mu_t$ is the memory unit. $mu_{t-1}$ is the memory unit of previous step. ${W_{ig}}$ is the input weight vector of the input gate. ${W_{fg}}$ is the input weight vector of the forget gate. ${W_{og}}$ is the input weight vector of the output gate. ${W_{mu}}$ is the input weight vector of the candidate unit. ${U_{ig}},{U_{fg}},{U_{og}},{U_{mu}}$ is the cyclic weight vectors of each gated unit. ${b_{ig}},{b_{fg}},{b_{og}},{b_{mu}}$ is the bias vector of each gated unit. tanh is the activation function. $\sigma$ $\cdot$ is the sigmoid function.

### Attention mechanism

The attention mechanism recognizes that specific regions require varying levels of focus at different times. This strategy enables prediction models to efficiently concentrate on crucial areas while reducing emphasis on less relevant regions. Redistributing attention between key and non-key regions improves model prediction accuracy^33,35,43^. To prevent LSTM networks from losing critical information due to extended temporal data, a probabilistic approach should be used instead of random weight allocation.

Attention mechanisms play a crucial role in Remaining Useful Life (RUL) prediction by allowing models to focus on the most significant aspects of the input data. By dynamically adjusting focus across different time steps, attention highlights features that have the greatest impact on the predicted RUL. This selective emphasis helps the model effectively capture long-term dependencies in time series data. Additionally, attention mechanisms enhance interpretability by revealing which input features drive the predictions. This transparency enables maintenance teams to better understand model behavior and make informed decisions based on identified critical parameters. Overall, attention mechanisms improve both prediction accuracy and the clarity of model results.

The attention mechanism relies on three key components: the query vector, key vectors, and value vectors. These elements are used to compute attention weights through score calculation:

20 $$\begin{aligned} score(q,k)=q \cdot k \end{aligned}$$

where, q is the query vector and k is the key vector. After that calculated Scaled Score:

21 $$\begin{aligned} score_{scaled}(q,k)=score(q,k) / \sqrt{d_k} \end{aligned}$$

where, $d_k$ is the dimension of the key vectors. Attention scores are normalized using the softmax function to obtain the weight coefficients for each part.

22 $$\begin{aligned} Attention(q,K,V)=softmax( score_{scaled}(q,k)) \end{aligned}$$

where, where K are all key vectors and V are all value vectors. The weight coefficients are utilized to execute a dot product operation with the known value vector V, yielding the attention value.

23 $$\begin{aligned} Attention(q,K,V)=\sum _i Attention(q,k_i) \cdot v_i \end{aligned}$$

where, $v_i$ value vector corresponding to the key vector $k_i$

### Fully connected layers

These layers are also known as dense layers. It connects every neuron in one layer to every neuron in the following layer^18,44^. The operation of it can be expressed mathematically as follows: In the forward pass, let an input vector x of size n and a fully connected layer with m neurons, the forward pass through the fully connected layer can be represented as: For each neuron j in the layer

24 $$\begin{aligned} z_j=\sum _{i=1}^{n} w_{ij} \cdot x_i + b_j \end{aligned}$$

where, $w_{ij}$ is the weight connecting the ith input neuron to the jth neuron in the layer, $b_j$is the bias term for the jth neuron. Besides, An activation function ReLU applied to the weighted sum to introduce non-linearity:

25 $$\begin{aligned} a_j=\sigma (z_j) \end{aligned}$$

where, $\sigma (\cdot )$ express the activation function.

### Theoretical analysis of CAELSTM’s generalization ability

The generalization ability of a machine learning model relates to its ability to perform effectively on previously unknown data. Factors influencing generalization in deep learning models include overfitting prevention, regularization, dataset variety, and noise robustness. The CAELSTM model combines numerous architectural choices to improve its generalization. The CAE component supports unsupervised feature extraction, allowing the model to focus on the most relevant degradation patterns while filtering out noise. By compressing the input data into a latent space representation, it reduces duplication and improves generalization across datasets. LSTM networks have the intrinsic ability to record long-term dependencies, which is critical in RUL prediction. However, typical LSTMs are susceptible to overfitting, particularly when trained on small datasets. To address this, our model incorporates dropout regularization into the LSTM layers to increase robustness. The inclusion of an attention mechanism further enhances generalization by ensuring the model focuses on the most significant degradation-related features. By dynamically adjusting weight distributions, attention mechanisms prevent the model from being biased toward irrelevant time steps, making it more adaptable to unseen operating conditions. To further improve the model’s generalization ability, Dropout layers included to prevent overfitting in the LSTM component.

## Experimental verification

### Data description

This study utilized NASA’s C-MAPSS (Commercial Modular Aero-Propulsion System Simulation), a tool designed for simulating turbofan engines, to generate the data. C-MAPSS simulates various operational, environmental, and control scenarios by adjusting input parameters^2^.

C-MAPSS includes run-to-failure data for 249 engines, simulated across six operational parameters. To create a more realistic environment, each of these engines has unique manufacturing differences and varying initial wear levels. The initial wear is attributed to differences in module efficiency. Engine failures occur due to one of two malfunction modes: High-Pressure Compressor (HPC) degradation or fan degradation. At the beginning of each time series, the engine operates normally, but a fault is introduced at some point, eventually leading to engine failure^2^.

A total of 21 sensors, installed across various engine modules, monitor the engine’s health. Table 2 provides a detailed list of all sensors. In addition to the 21 sensor readings, three extra parameters are recorded to reflect the engine’s operating conditions. Table 3 lists these operational parameters. All sensor and operational data are recorded once per engine cycle.

Table 4 summarizes key details of the C-MAPSS dataset, which consists of four sub-datasets—FD001, FD002, FD003, and FD004—each featuring different operational conditions and failure modes. Each sub-dataset is divided into training and test sets. The dataset contains 26 columns per row: the first five columns include the engine unit number, degradation time step, and operational settings, while the remaining 21 columns contain multivariate time-series data from the 21 sensors in the same operating cycle. As shown in Table 2, the sensor data include measurements of temperature, pressure, and speed, with additional details available in^4^.

Furthermore, this sensor data captures the engine’s progression from normal operation to initial wear and eventual failure. In this study, the proposed model is used to analyze the training dataset and predict the Remaining Useful Life (RUL) of engines in the test dataset.

**Table 2:** The description of sensors and their units.

Sensor number	Symbol	Description	Unit of measure
S1	T2	Total temperature at fan inlet	Rankine($^{\circ }$R)
S2	T24	Total temperature at LPC outlet	Rankine($^{\circ }$R)
S3	T30	Total temperature at HPC oulet	Rankine($^{\circ }$R)
S4	T50	Total temperature at LPT outlet	Rankine($^{\circ }$R)
S5	P2	Pressure at fan inlet	Pounds per square inch absolute(PSIA)
S6	P15	Total pressure in bypass-duct	Pounds per square inch absolute(PSIA)
S7	P30	Total pressure at HPC outlet	Pounds per square inch absolute(PSIA)
S8	Nf	Physical fan speed	Revolution per minute (rpm)
S9	Nc	Physical core speed	Revolution per minute (rpm)
S10	epr	Engine pressure ratio (P50/P2)	Nil
S11	Ps30	Static pressure at HPC outlet	Pounds per square inch absolute (PSIA)
S12	phi	Ratio of fuel flow to Ps30	pps/psi
S13	NRF	Corrected fan speed	Revolution per minute (rpm)
S14	NRc	Corrected core speed	Revolution per minute (rpm)
S15	BPR	Bypass Ratio	Nil
S16	farB	Burner Fuel-air ratio	Nil
S17	htBleed	Bleed Enthalpy	Nil
S18	NF_dmd	demanded fan speed	Revolution per minute (rpm)
S19	PCNfR_dmd	demanded corrected fan speed	Revolution per minute (rpm)
S20	W31 HPT	HPT coolant bleed	Ibm/s
S21	W32 LPT	LPT coolant bleed	Ibm/s

**Table 3:** Operational parameters.

Operational parameter	Description
Tr	Throttle resolver angle (TRA)
Al	Altitude
MN	Match number

**Table 4:** C-MAPSS datasets description.

Datasets	FD001	FD002	FD003	FD004
No. of engines for training	100	260	100	249
No. of engines for testing	100	259	100	248
Operation conditions	1	6	1	6
Fault mode number	1	1	2	2
Training sample	20,631	53,759	24,720	61,249
Test sample	13,096	33,991	16,596	41,214

### Performance metrics

The system’s RUL prediction model utilizes multisensor monitoring data to forecast aircraft engine degradation, with the predicted RUL as the output. To evaluate the effectiveness of different techniques, three metrics are used: Root Mean Squared Error (RMSE), Mean Absolute Error (MAE), and a scoring function.

1. Root mean square error (RMSE)^19^ is a widely used metric for evaluating the performance of regression models. It measures the average magnitude of errors between predicted and actual values in a dataset. Here’s a simple explanation:
  26 $$\begin{aligned} RMSE_R{EMPTY}_U{EMPTY}_L=\sqrt{(1/N) \sum _{i=1}^{N}(Y_i - \hat{Y_i}^2)} \end{aligned}$$
  where: N represents the total number of test data samples. $Y_i$ is the true value of the engine life. $\hat{Y_i}$ is the predicted value of the engine life.
2. Mean absolute error (MAE) is known as the loss function, which is derived by averaging the absolute differences between the predicted and actual target values. It is represented as follows:
  27 $$\begin{aligned} MAE_R{EMPTY}_U{EMPTY}_L=\sqrt{(1/N) \sum _{i=1}^{N}|Y_i - \hat{Y_i}|} \end{aligned}$$
  where: N represents the total number of test data samples. $Y_i$ is the true value of the engine life. $\hat{Y_i}$ is the predicted value of the engine life. $|-|$ denotes the absolute value. It is often used as a loss function, particularly in regression problems aimed at predicting continuous values. During model training, the objective is to minimize the MAE loss, which represents the average absolute difference between the predicted and actual values.
3. Scoring Function^18^ is often defined as a function that evaluates a model’s performance on a specific dataset. Scoring functions are used to assess a model’s ability to predict or classify data. It represents the average value of the test set fraction. A lower score indicates better performance. The calculation formula is:
  28 $$\begin{aligned} Score= {\left\{ \begin{array}{ll} \sum _{i=1}^{N} (e^{\frac{dif_i}{13}} - 1), & \text {if } dif_i < 0 \\ \sum _{i=1}^{N} (e^{\frac{dif_i}{10}} - 1), & \text {if } dif_i \ge 0 \end{array}\right. } \end{aligned}$$

**Fig. 3:**
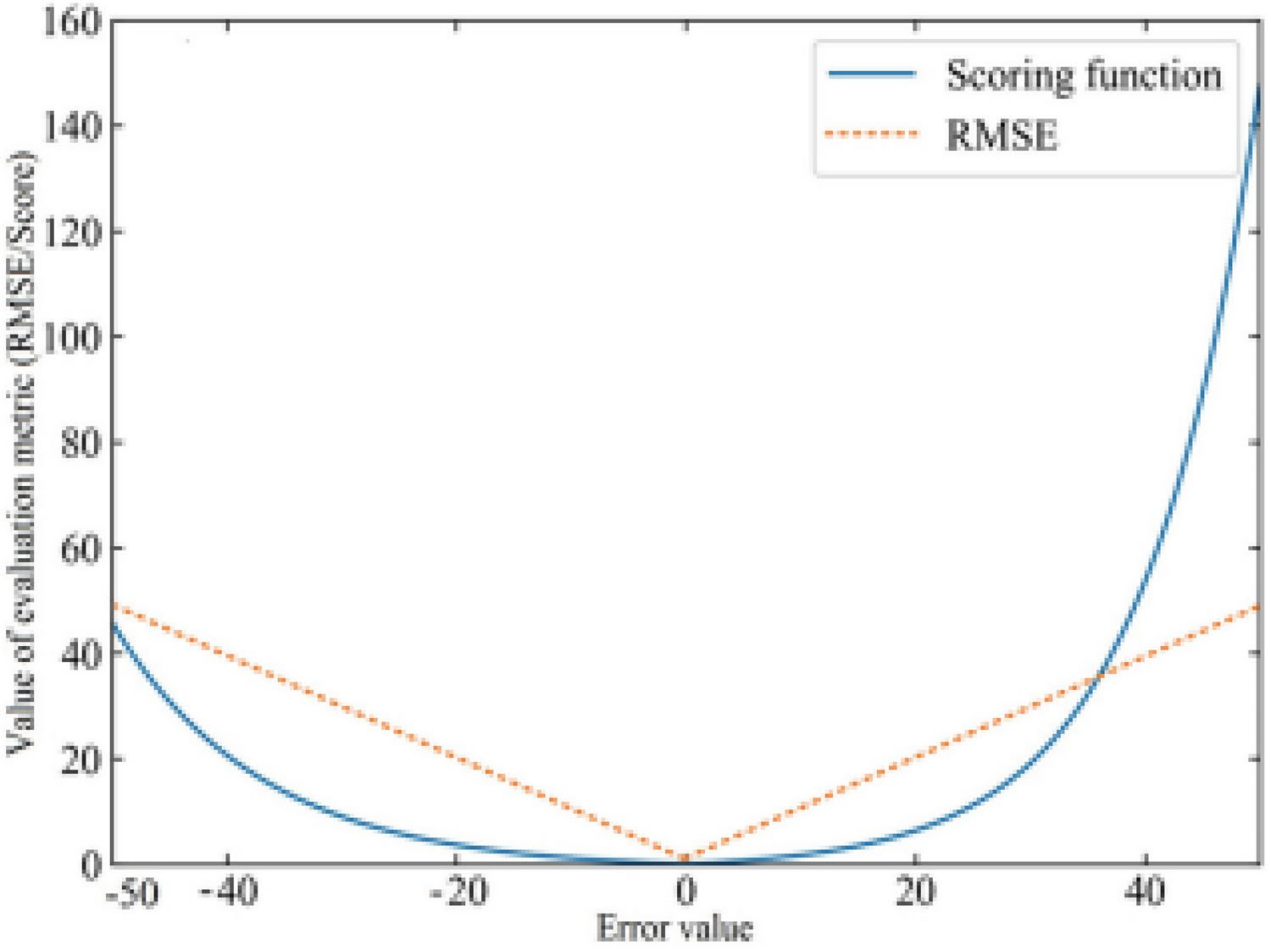
RMSE versus scoring function (Fig. 7 in^33^).

When employing deep learning models to predict RUL, Eq. 28 shows that the scoring function penalizes late predictions more heavily, whereas RMSE treats both early and late predictions equally. Late predictions are considered more critical as they can lead to severe accidents. Figure 3 illustrates the comparison between RMSE and the scoring function. It demonstrates that the penalty for late predictions is significantly higher than for RMSE, emphasizing the goal of preventing engine failures. The linear relationship between RMSE and error values suggests that RMSE has a well-defined physical meaning. The model’s predictive capability was evaluated using both RMSE and the scoring function, as they serve as crucial performance indicators.

### Data preprocessing

#### Sensor selection

All of our analysis is based on sensor measurements. The first two columns of the training data contain engine and cycle information, which are not required for training the algorithm. Similarly, columns 3, 4, and 5 represent operating settings, which we will also exclude from training. It is worth noting that some researchers use operational setting values in their algorithms, but we will not.

**Fig. 4:**
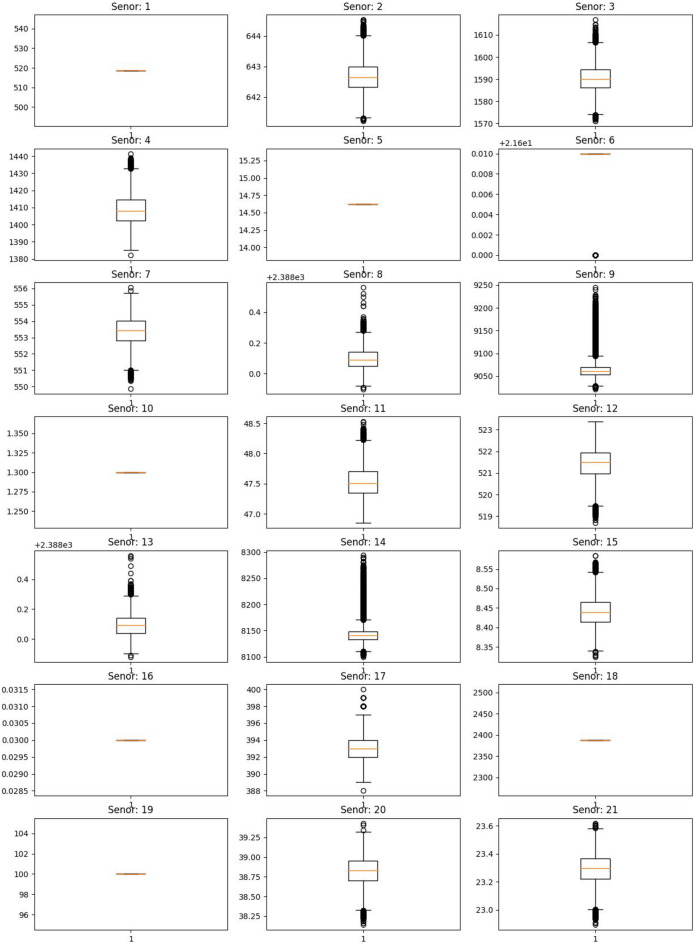
Boxplot of sensor measurements in FD001 dataset.

We will train our algorithm solely using the values from columns 6 to 26, as they contain sensor measurements. Figure 4 presents boxplots of all sensor measurements from the FD001 training dataset.

From Fig. 4,we observe that sensors 1, 5, 10, 16, 18, and 19 have constant values. Additionally, sensor 6 appears to have only a few distinct values^33^. Specifically, sensor 6 has two constant values: 21.61 and 21.60, with 21.61 occurring 20,225 times. Since these values are extremely close together, we can consider the entire column to be constant.

Constant values are not particularly useful for training algorithms. Moreover, normalizing data is sometimes necessary, but if a column has a constant value, its standard deviation becomes 0. As a result, normalization is impossible due to division by zero. Therefore, we will remove columns with constant values.

The selection of sensor measurements was conducted after a thorough investigation of feature importance. Some sensors exhibited little or no correlation with the degradation process, indicating that their inclusion would not significantly enhance prediction performance.

**Fig. 5:**
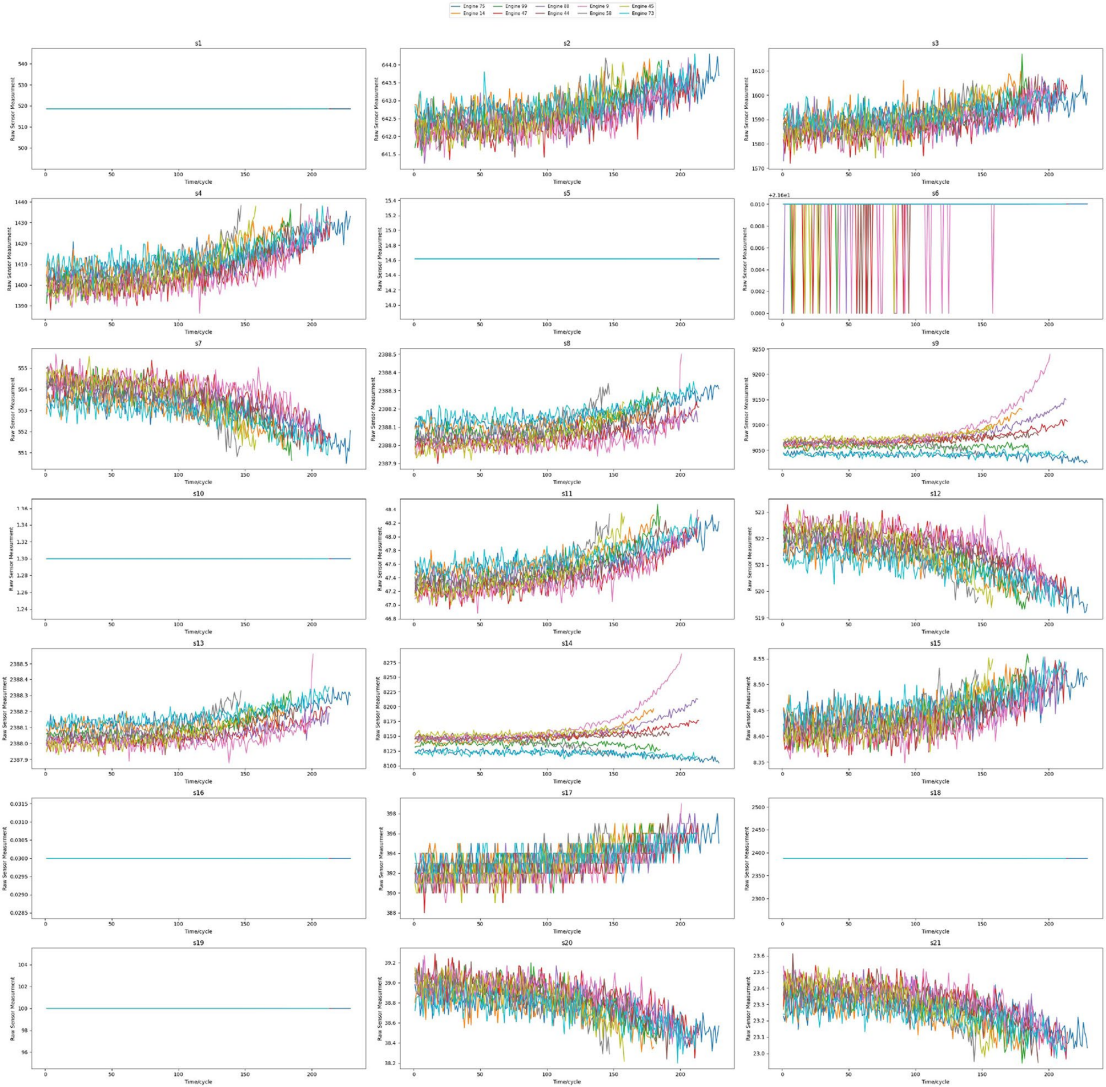
Sensor degradation measurements over Time for random selected engines.

Figure 5 illustrates sensor measurements over time for a subset of randomly selected engines from the FD001 training data.

#### Data normalization

Data normalization is primarily considered the first step in deep learning. Thus, to retain data on the same scale, the dimensions of multiple sensors must be standardized^45^. We use a StandardScaler approach (Z-score) to normalize the input data, ensuring it falls within the range of 0 to 1. The formula for Z-score is given by:

29 $$\begin{aligned} Z=\frac{x-\mu }{\sigma } \end{aligned}$$

where: z represents the standardized data. x represents the original value of the feature. $\mu$ is the mean value of the feature. $\sigma$ is the standard deviation of the feature.

**Fig. 6:**
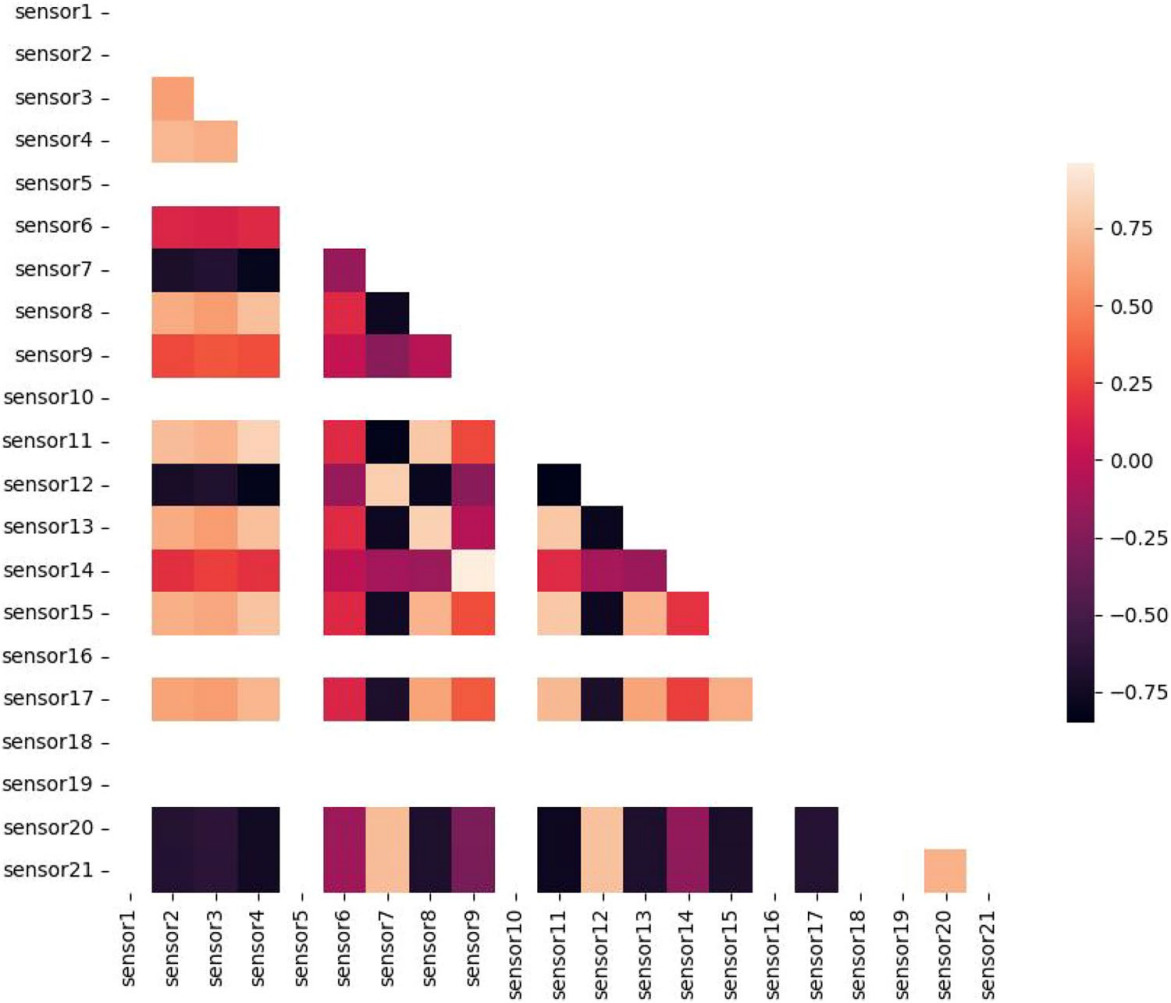
Heatmap of all sensors in dataset FD001.

**Fig. 7:**
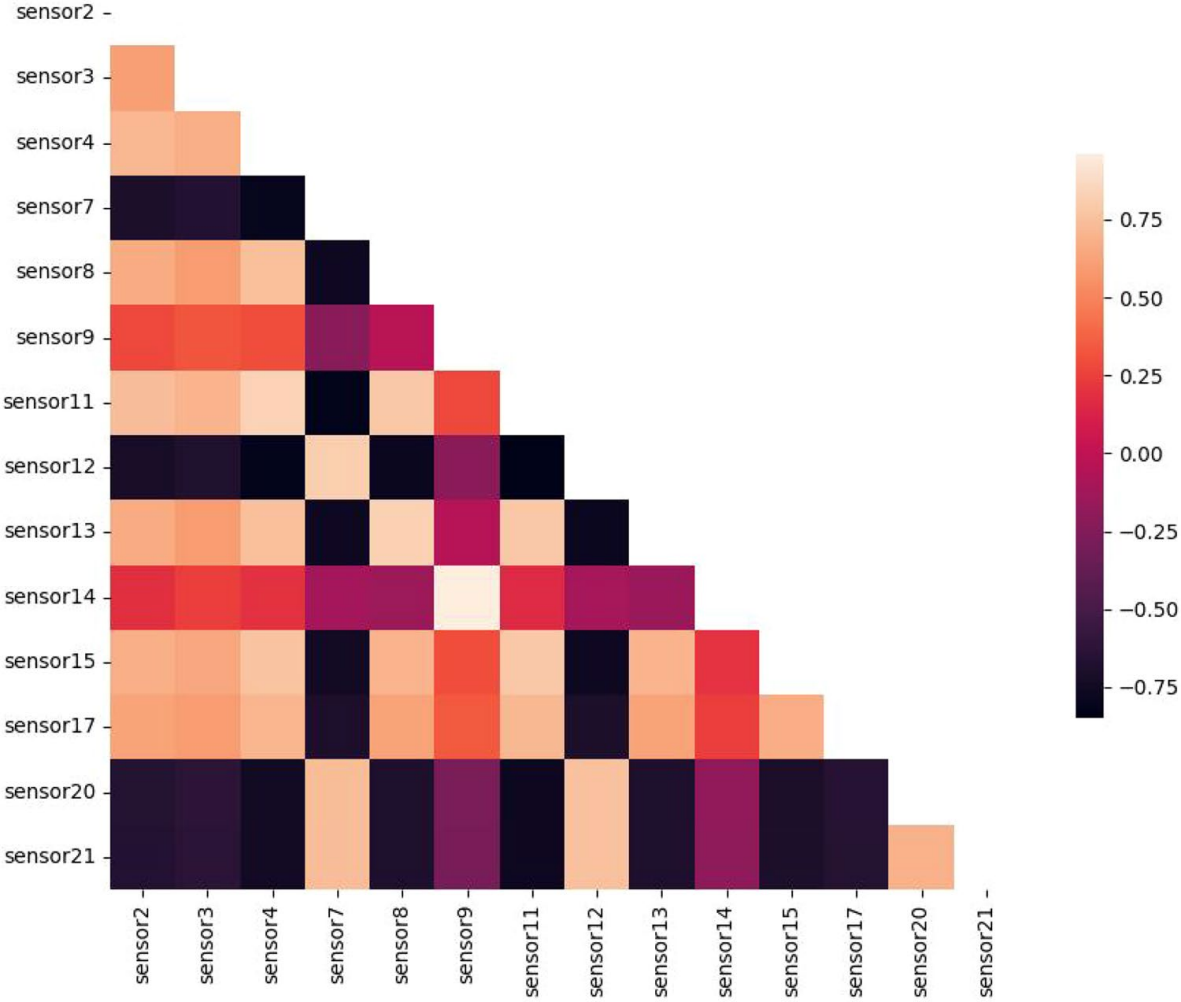
Heatmap of all sensors without sensor 1, 5, 6, 10, 16, 18 and 19.

Correlation analysis of the sensors in dataset FD001 is presented in Fig. 6. From this figure, we can see the obvious relationships between all sensors in the dataset. Figure 7 presents the correlation analysis without the excluded sensors mentioned in the data selection.

Normalization is crucial in RUL prediction since it scales the input characteristics to a constant range, which helps the model’s convergence speed during training. It reduces the impact of different magnitudes of sensor readings, allowing the model to learn significant patterns more efficiently. Furthermore, it improves prediction accuracy and model generalization by ensuring that all features contribute equally.

**Fig. 8:**
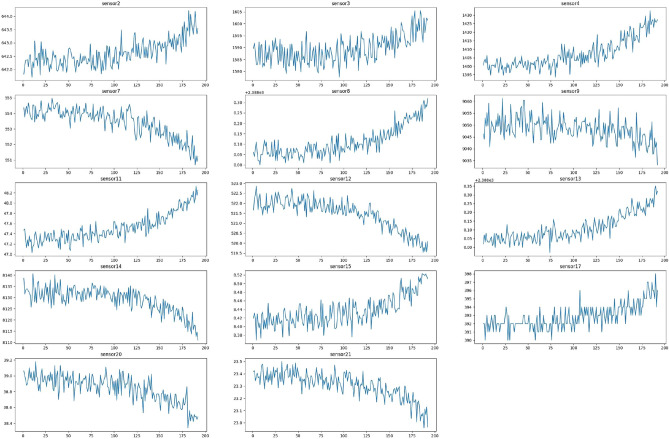
Raw sensor data before normalization.

**Fig. 9:**
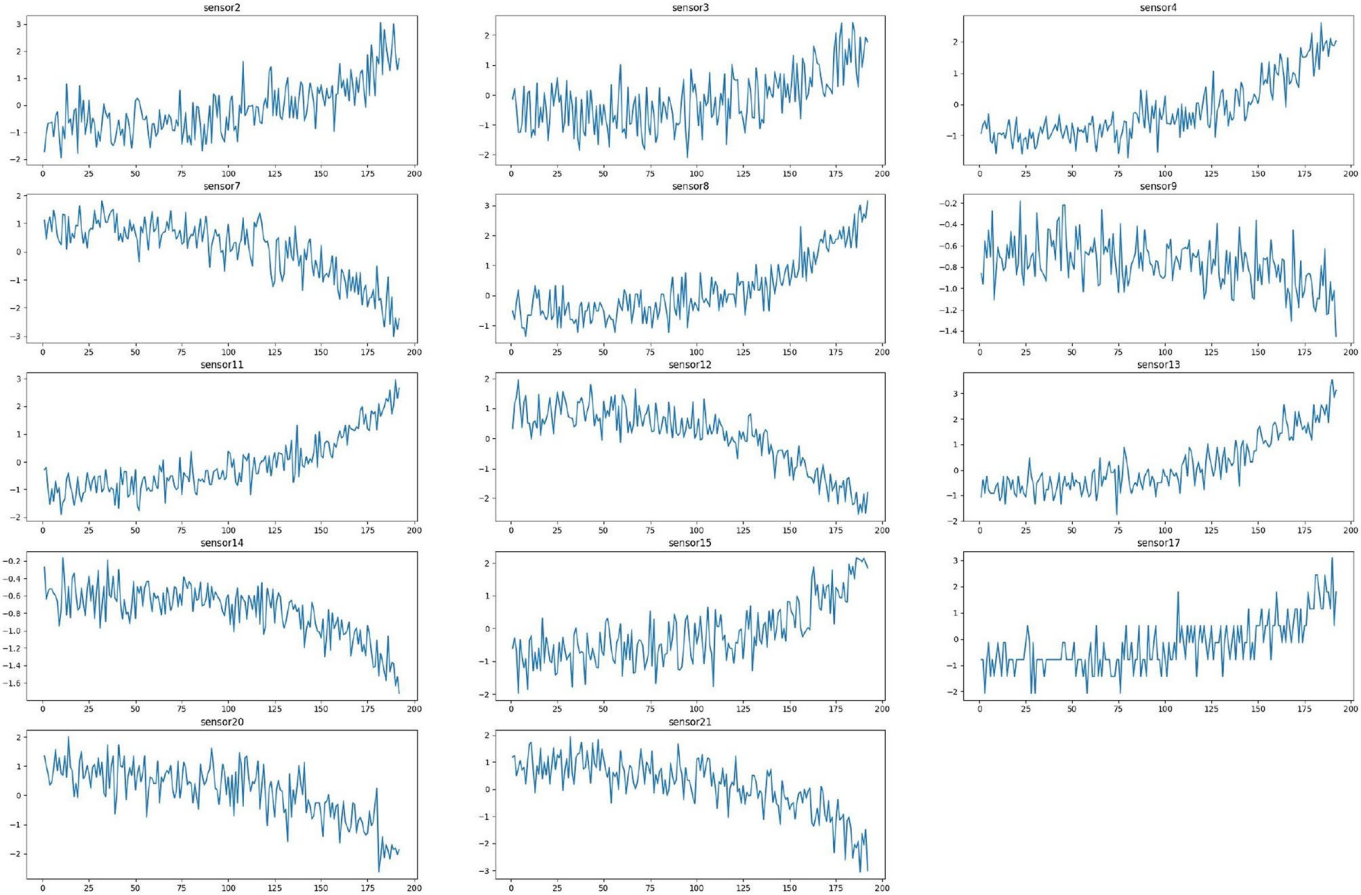
Standardized sensor data.

It can be seen from Fig. 8 data signal of sensors before standardization and the standardized sensor data signal in Fig. 9.

#### Piecewise degradation model

To accurately anticipate RUL labels, it’s important to analyse the turbofan engine’s specific features. During regular use, the engine’s lifespan decreases in a predictable manner. To improve model prediction accuracy, it is assumed that the engine does not degrade during its first operation and has a constant lifetime. When the engine’s RUL reaches the turning point of lifetime decay, it begins to degrade linearly, resulting in its real lifetime^33^.

In dataset FD001 there is no RUL data provided for the training set. It is easy to calculate RUL values directly from training data. Taking into account that training data includes run-to-failure data for all engines. Engine 1 fails at 192 cycles. So, during the first cycle of engine 1, the RUL is 191. After cycle two, the RUL is 190, and so on. It fails after 192 cycles, therefore its RUL is 0. This is known as a linear degradation model. It gradually diminishes from the beginning of the cycle until it reaches zero at the end. In addition to the linear degradation model, the piecewise linear degradation model is also commonly employed. In this concept, RUL is initially allocated to a fixed number (for a number of cycles). The fixed number is known as early RUL. When the RUL value hits the early RUL, it then follows a linear decline paradigm.

Piecewise linear degradation models are more flexible in reflecting different degradation patterns over time than exponential models, which assume a constant rate of decrease. They enable for more rapid changes in degradation behaviour, better representing real-world conditions. These models enhance interpretability, making it easier to identify critical operating stages. Furthermore, piecewise linear models are resistant to noise in sensor data, which reduces the danger of overfitting. When combined with CAELSTM, they can improve prediction accuracy by learning local trends and deterioration transitions. This results in more accurate maintenance planning and decision-making^35^.

**Fig. 10:**
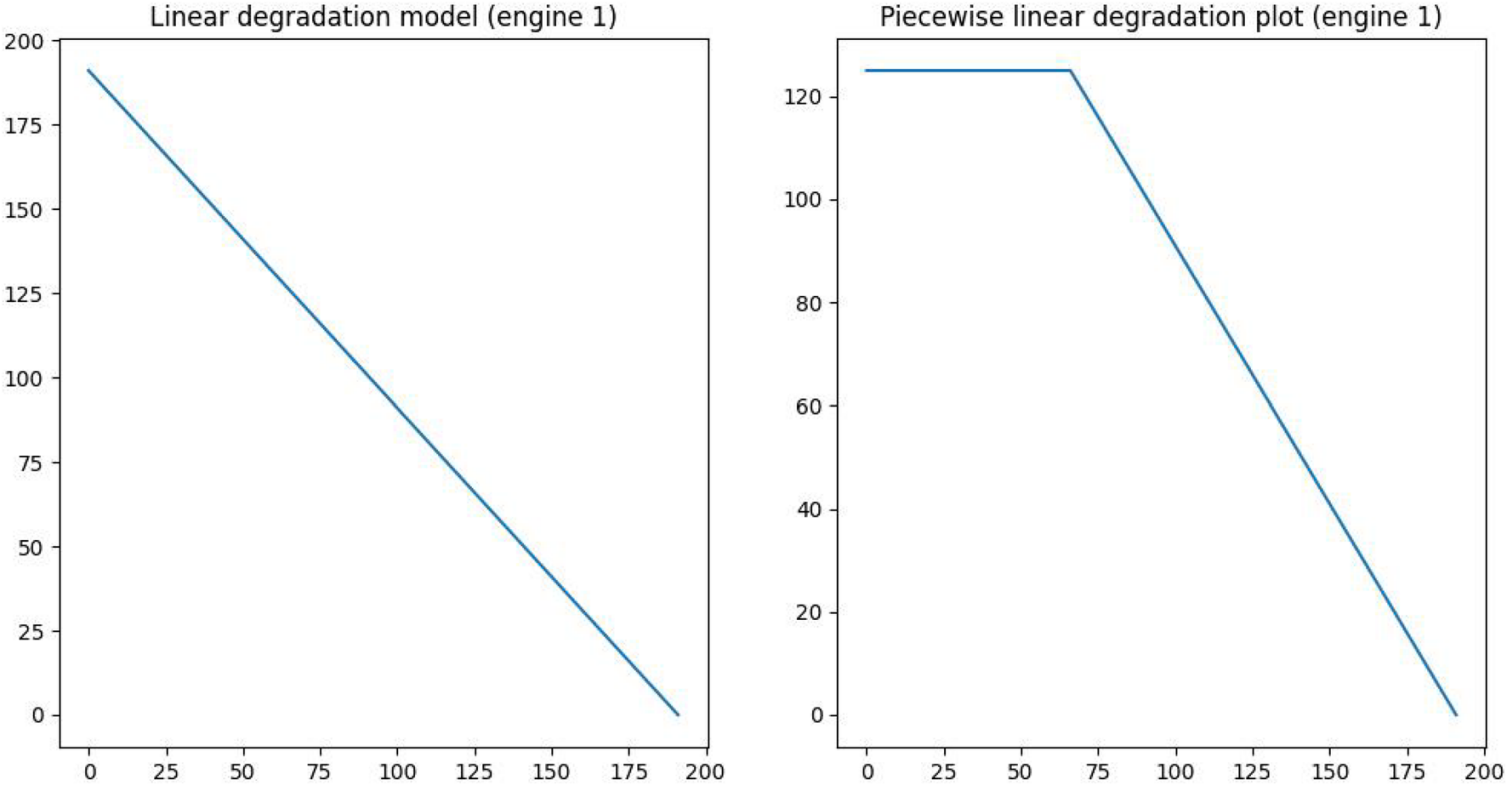
Difference between linear degradation and piecewise linear model.

**Fig. 11:**
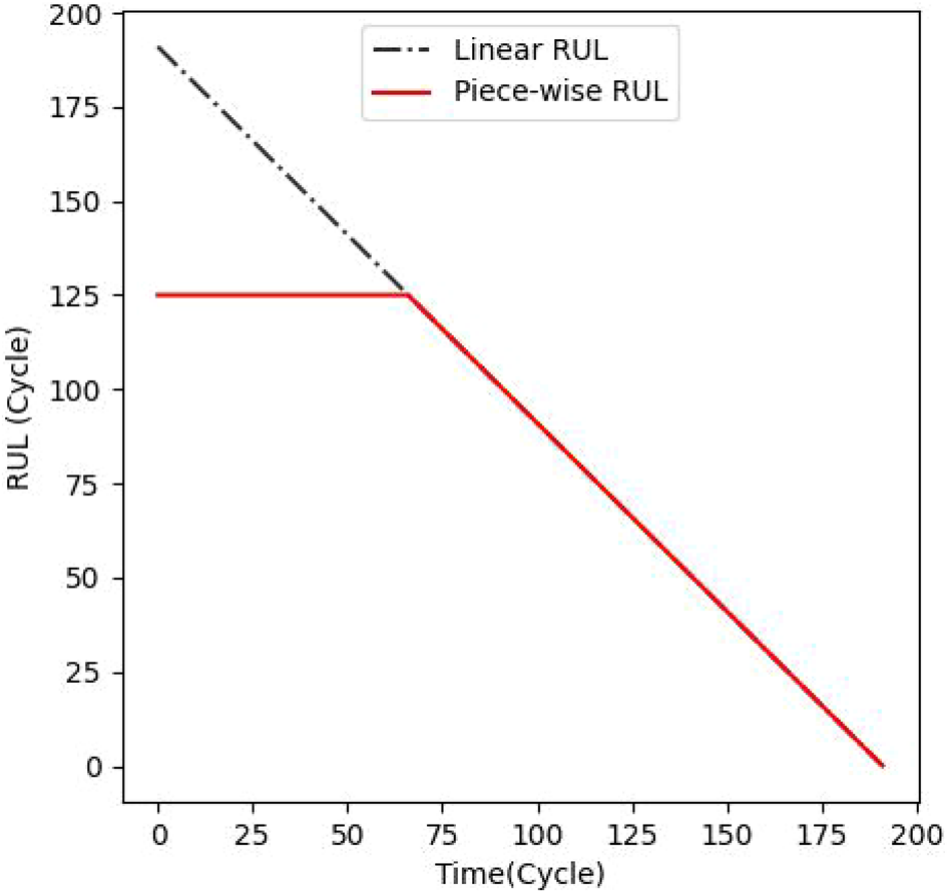
Piecewise linear degradation model.

To demonstrate both degradation models, we shall present RUL values for engine 1 using both models in Fig. 10.The RUL of engine 1 is 192 (as mentioned above). For the piecewise linear degradation model, we set the early RUL to 125 according to the study^46^. There is no set rule for selecting this value. Figure 11 shows that the RUL label degrades linearly with time, eventually failing completely.

#### Time window processing

**Fig. 12:**
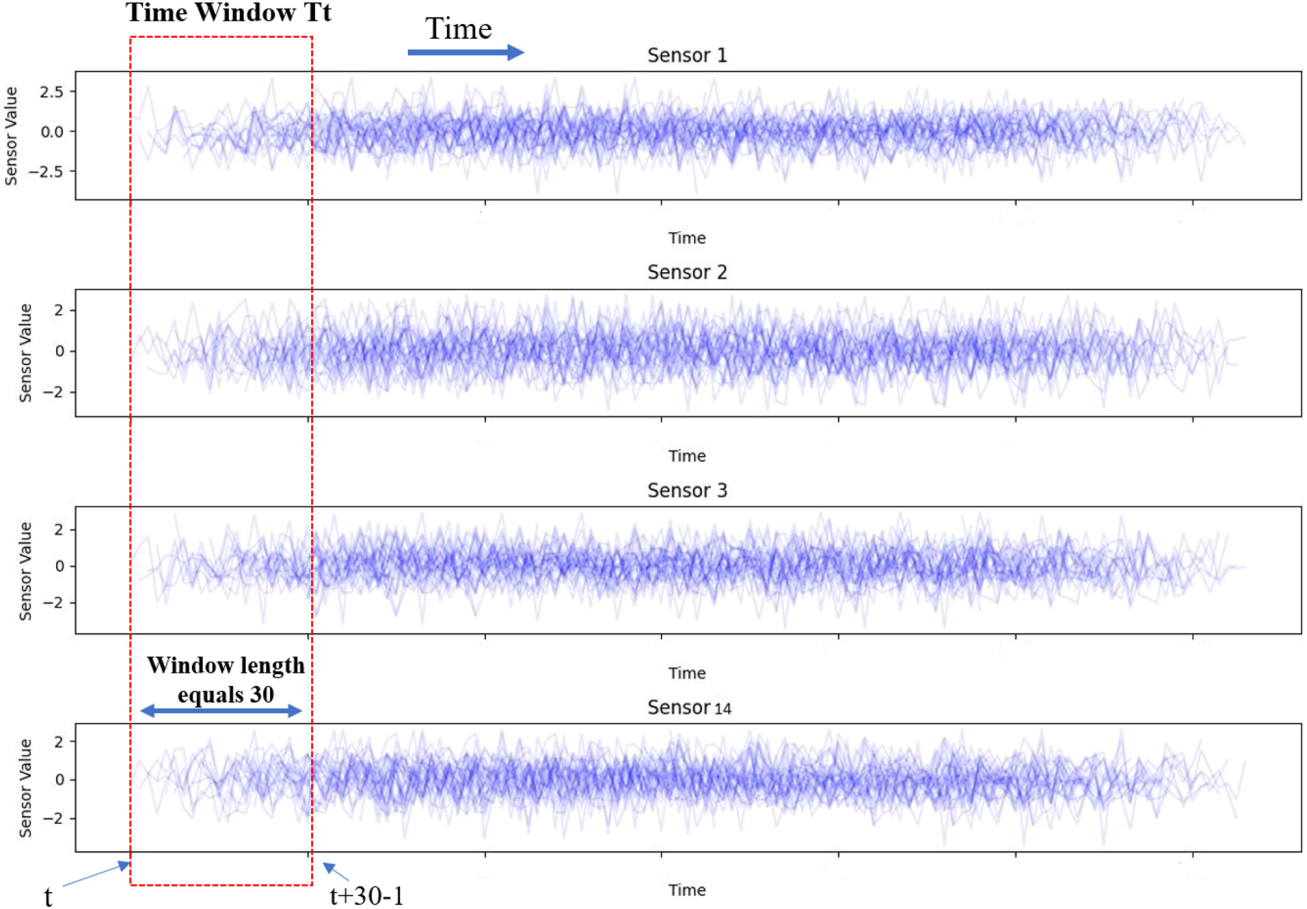
Time window processing.

Time window processing is a data improvement technique. Sliding window processing captures the dependencies between time series data^33^. Figure 12 depicts the time window processing. Set the sliding time step to 1 and use the previous time step to forecast the next. The length of the window depends on the original data. Longer time windows offer more valuable information. The length of the time window should not exceed the minimal life cycle. To achieve satisfactory results, we choose an appropriate window length for FD001 and FD003. The minimum life cycle of training subsets is 128 and 145 respectively. The minimum life cycle of testing subsets is 31 and 38 respectively. the time window size is equal 30 this is shown in Table 5. The window size is determined through experimental design. The experiment try window sizes of 30, 35, 40, and 45. when the window size is 30, the proposed model can obtain the best results. Therefore, 30 is selected as the window size.

**Table 5:** Time window for two subsets FD001 and FD003.

Subsets	Min life cycle in training datasets	Min life cycle in testing datasets	Time window size
FD001	128	31	30
FD003	145	38	30

### Experimental analysis and results

#### Experimental setting and description

The training and testing procedure uses Tensorflow 2.16.1, Numpy 1.26.4, Pandas version 2.2.3 and Scikit-learn 1.2.2. The hardware platform includes an Intel(R) Core(TM) i7, 8 GB of RAM, and Windows 10 as the operating system. A Kaggle Platform is used for speeding up the processing of deep learning computation. We run all of our experiments on it to use it’s capabilities. Table 6 lists the suggested model’s hyper-parameters.

**Table 6:** Model hyper-parameters.

Parameters	FD001	FD003
size	512	32
Training rounds	25	25
Activation Function	relu	relu
CNN Layers	3	3
LSTM Layers	1	1
Attention neurons	1	1
Learning rate	0.001	0.0001
convolutional kernel size	3	3
Dropout	0.4	0.6
Optimizer	Adam	Adam
Loss	Mean squared error (MSE)	Mean squared error (MSE)

**Fig. 13:**
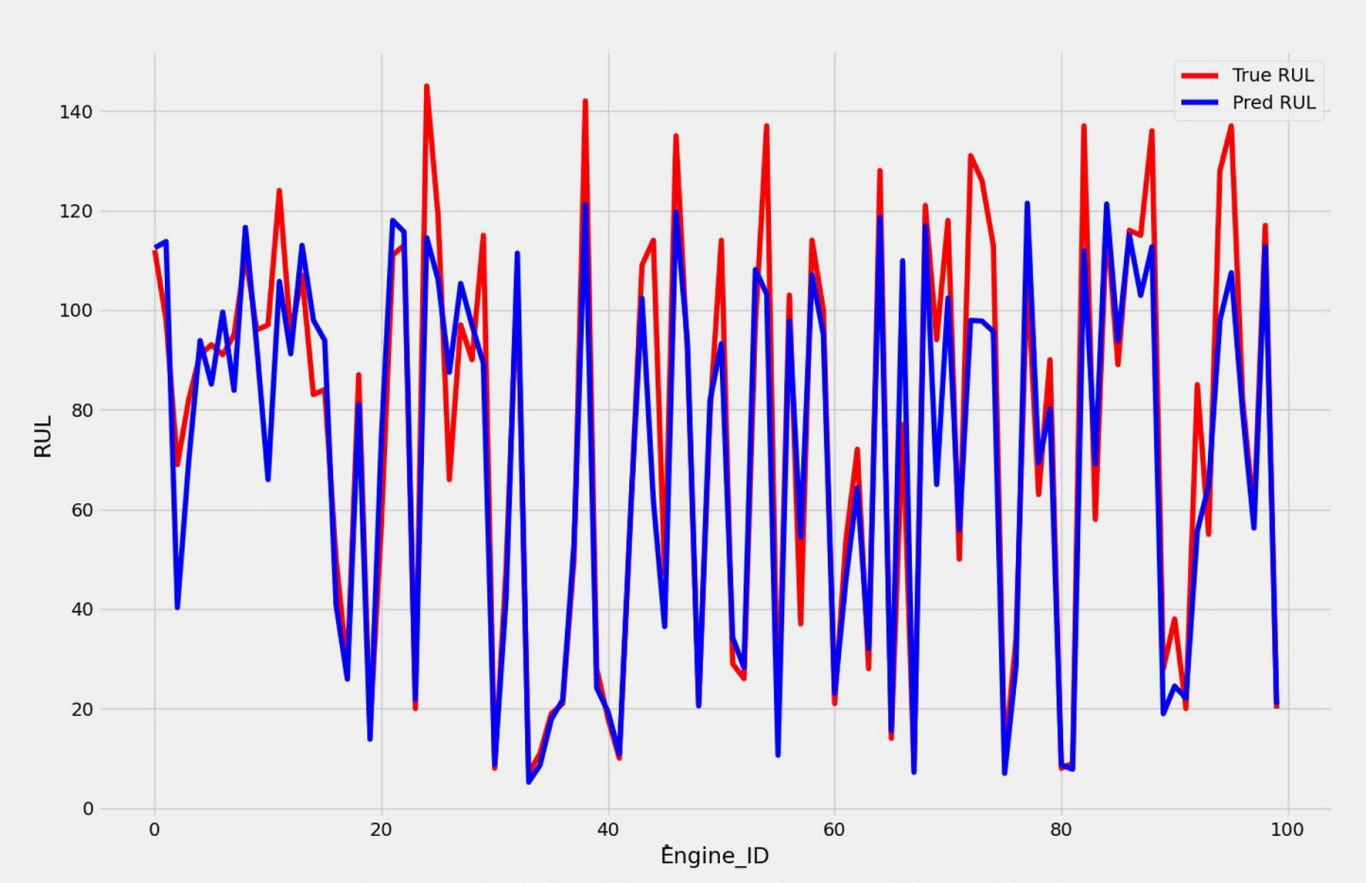
RUL prediction results for FD001.

**Fig. 14:**
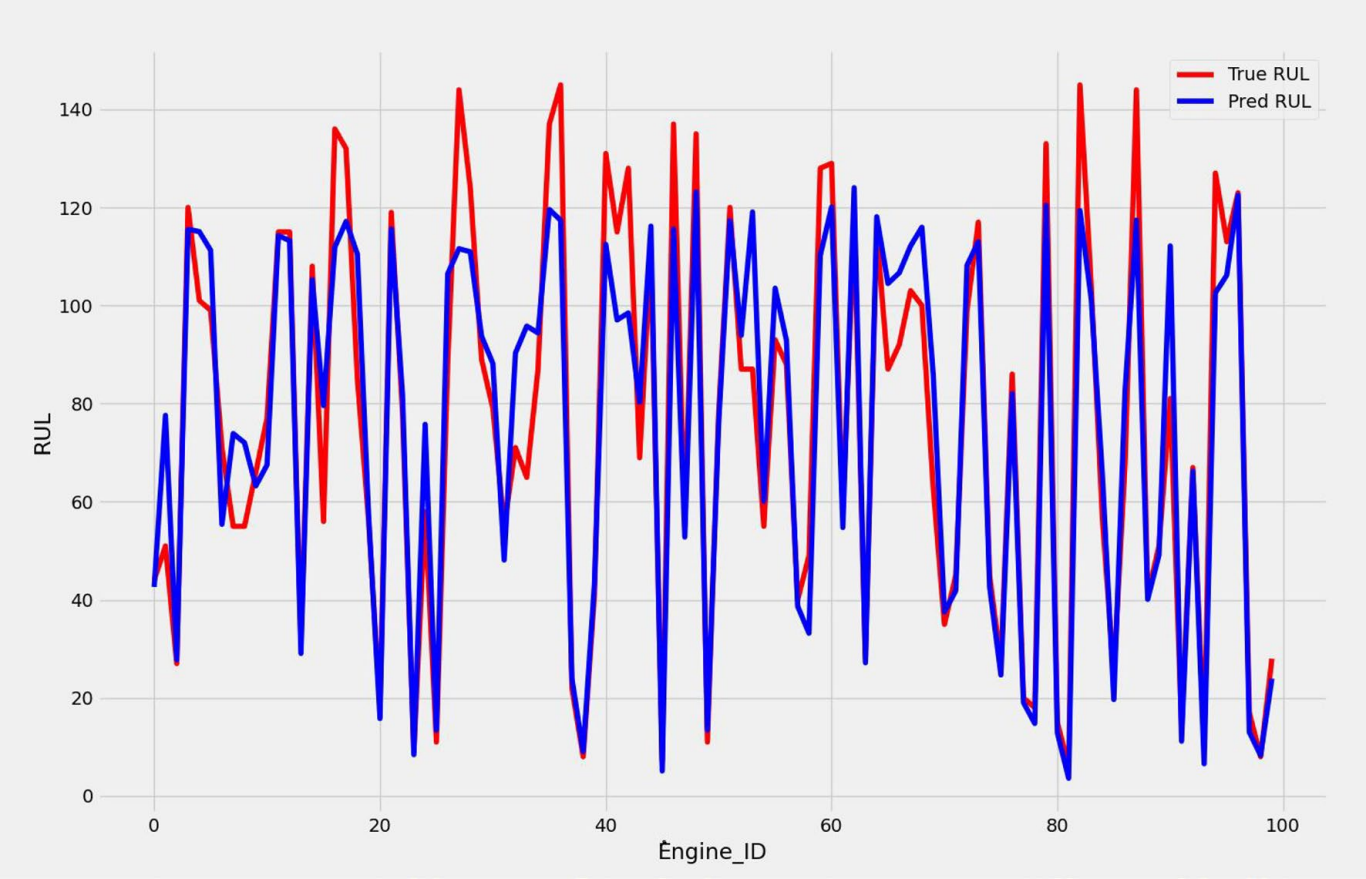
RUL prediction results for FD003.

We present a model based on convolutional autoencoders, LSTMs, and an attention mechanism to predict the RUL of aero-engines. Figures 13 and 14 show the predicted RUL values for 100 engine units from the FD001 and FD003 sub-datasets alongside the actual RUL values. The results demonstrate that the proposed model’s predictions closely match the actual values.

**Fig. 15:**
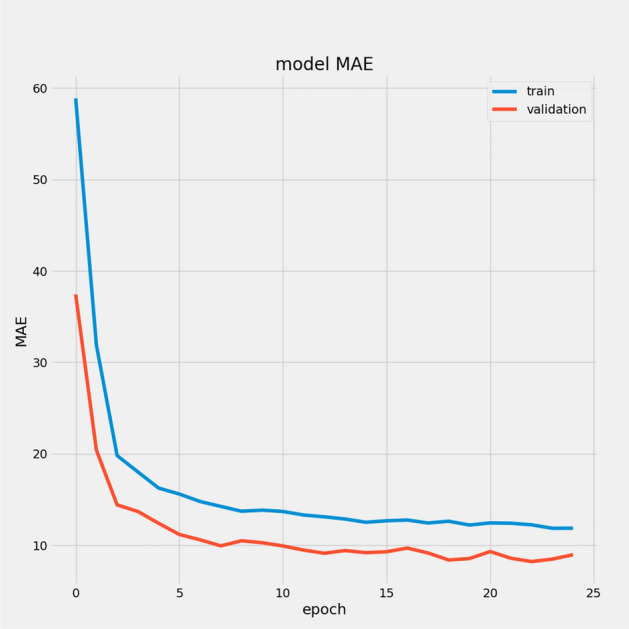
Model MAE for FD001.

**Fig. 16:**
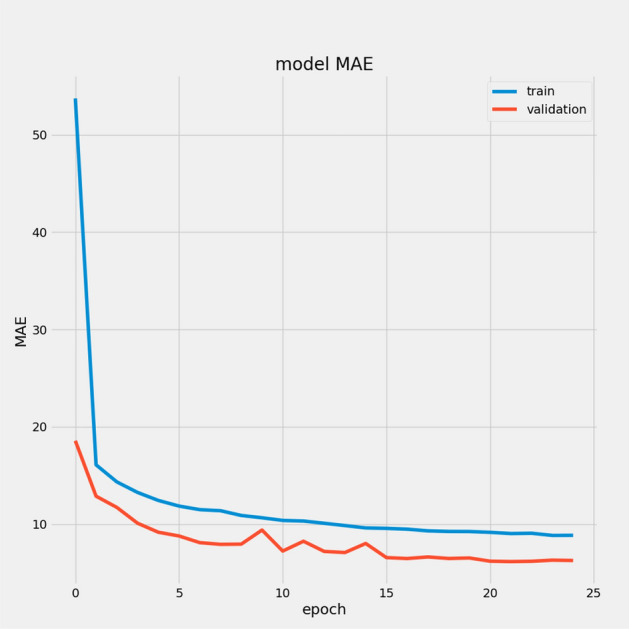
Model MAE for FD003.

**Fig. 17:**
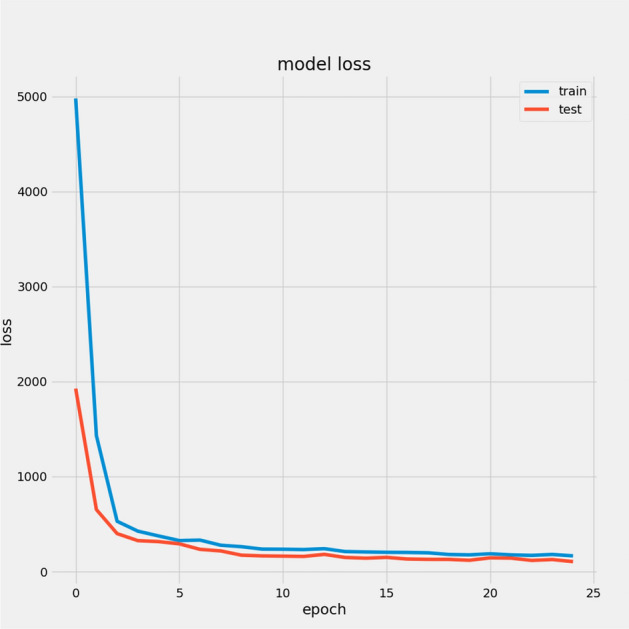
Model Loss For FD001.

**Fig. 18:**
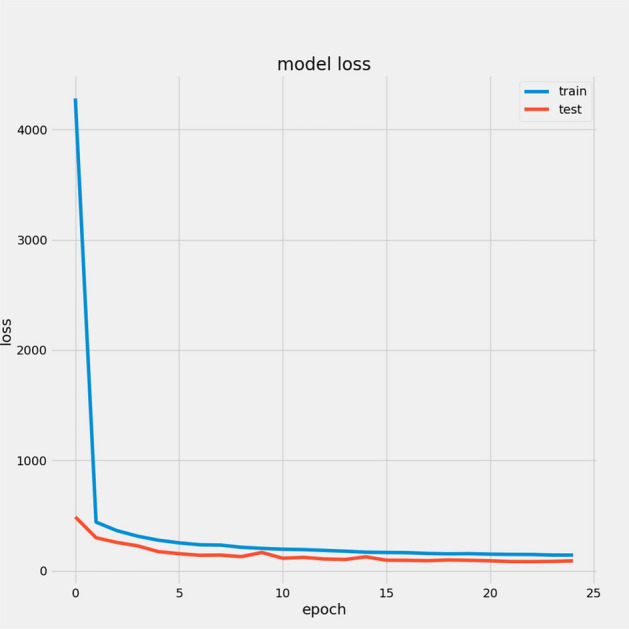
Model Loss For FD003.

In Table 7, we report the RMSEs for the proposed model with the two datasets: 14.44 for FD001 and 13.40 for FD003; MAEs are 10.49 and 10.68, respectively, with scores of 282.38 and 264.47. During training, we calculated the Mean Absolute Error (MAE) and loss for both training and validation sets to assess model performance and monitor for overfitting or underfitting. Figures 15 and 16 illustrate the performance accuracy of the proposed model across the two sub-datasets, indicating an ideal range for model complexity. Figures 17 and 18 show that the training and validation loss values align well over 25 epochs, confirming that the model’s predictions are consistent with actual data, with no signs of overfitting or underfitting.

**Table 7:** Proposed model performance results on FD001 and FD003.

Subsets	FD001	FD003
RMSE	14.44	13.40
MAE	10.49	10.68
Score	282.38	264.47

#### Impact of learning rate

**Fig. 19:**
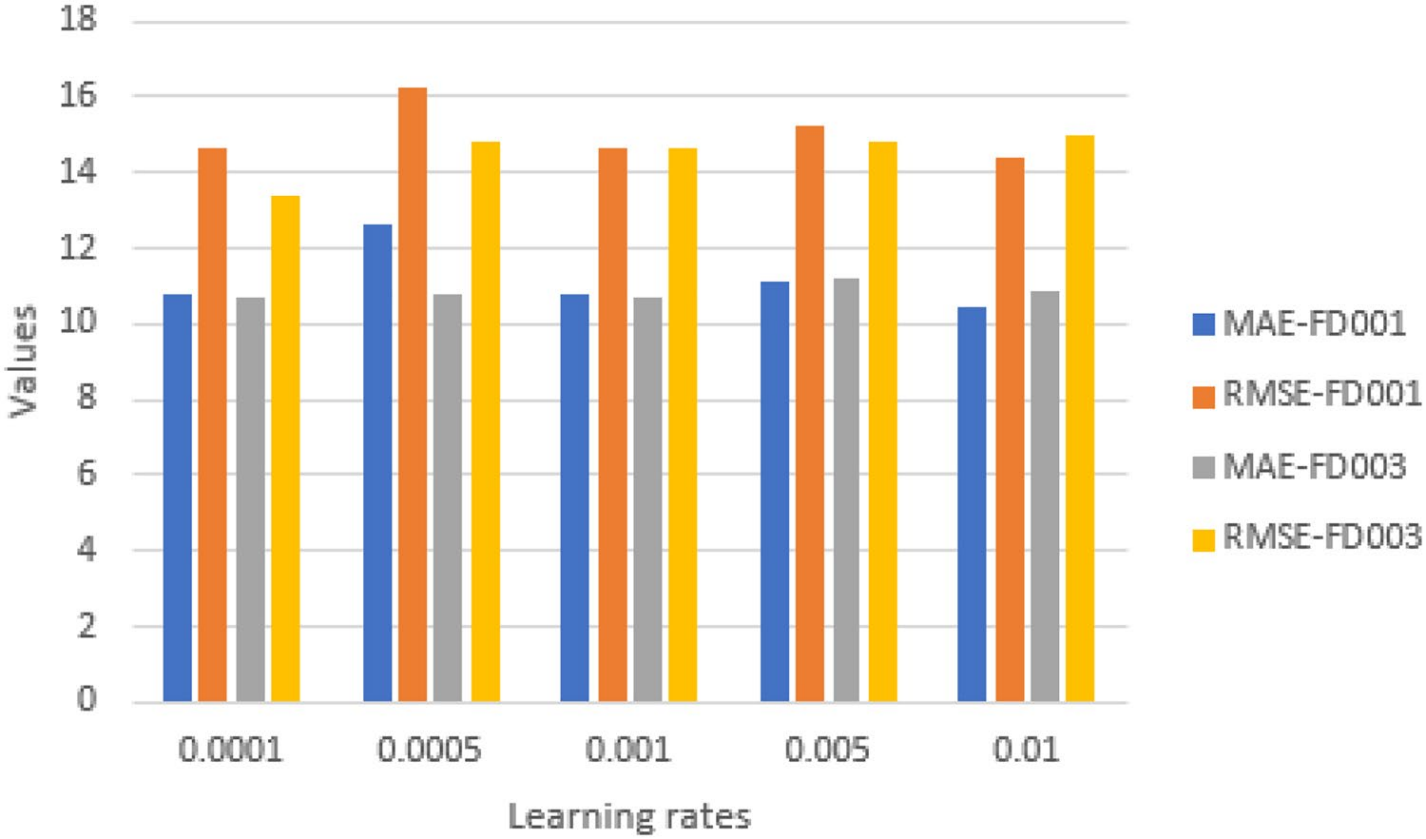
Impact of learning rate on model performance on FD001 and FD003 (MAE and RMSE).

**Fig. 20:**
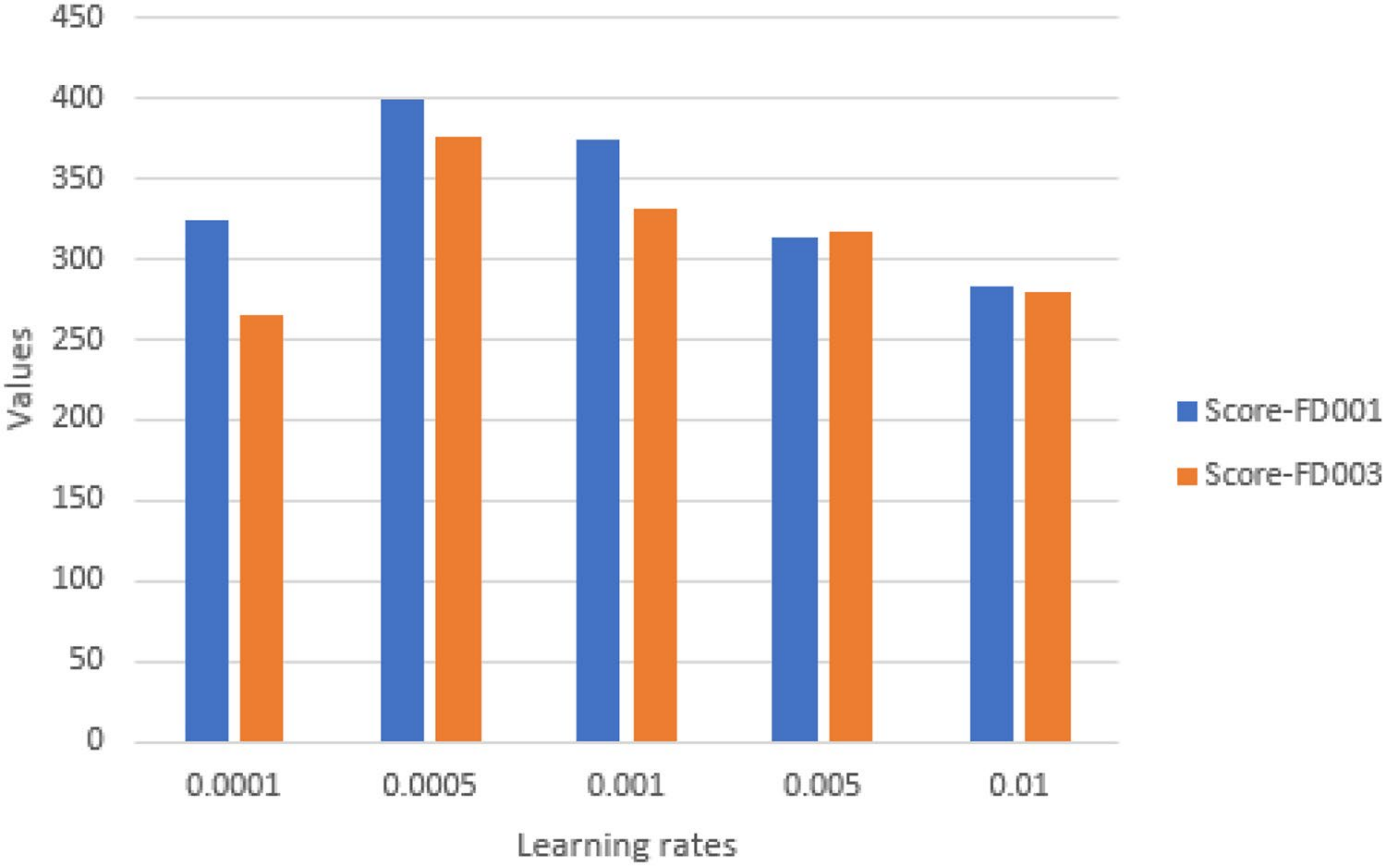
Impact of learning rate on model performance on FD001 and FD003 (scoring function).

It is a crucial hyper-parameter in deep learning, affecting the model’s ability to reach the local minimum within a reasonable time frame. In experiments we included learning rates of 0.0001, 0.0005, 0.001, 0.005, and 0.01. All other model parameters stay unaltered. Figures 19 and 20 display the results For FD001 and FD003. The proposed model performs optimally at a learning rate of 0.001 for FD001 and 0.0001 For FD003 (minimum RMSE, MAE, and score).Therefore, two values were chosen as the ultimate learning rates.

#### Impact of batch size

**Fig. 21:**
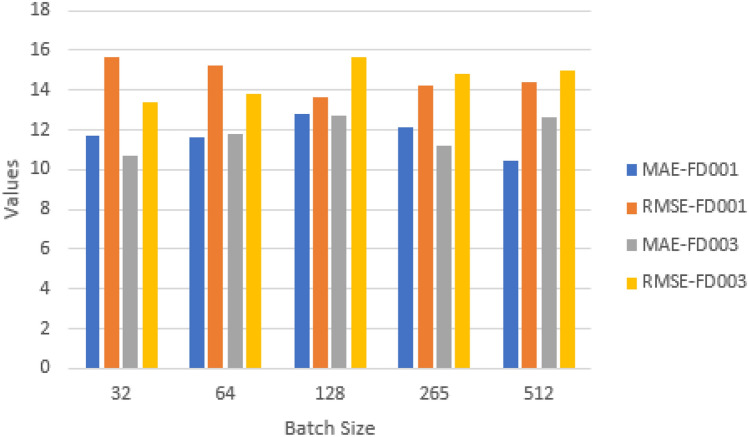
Impact of batch size on model performance on FD001 and FD003 (MAE and RMSE).

**Fig. 22:**
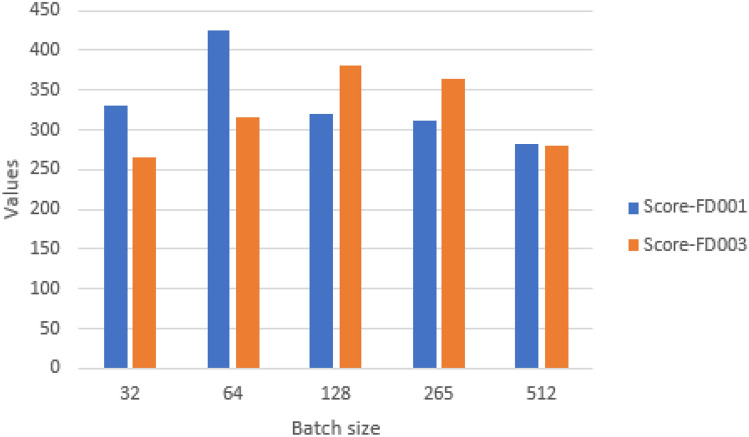
Impact of batch size on model performance on FD001 and FD003 (scoring function).

It determines the quantity of training data and impacts model speed and optimisation. In experiments we included batch sizes of 32, 64, 128, 256, and 512. Figures 21 and 22 show that with a batch size of 512 (FD001) and 32 (FD003), the proposed model yields the best results. Therefore, two values are chosen as the ultimate batch size.

#### Impact of dropout

**Fig. 23:**
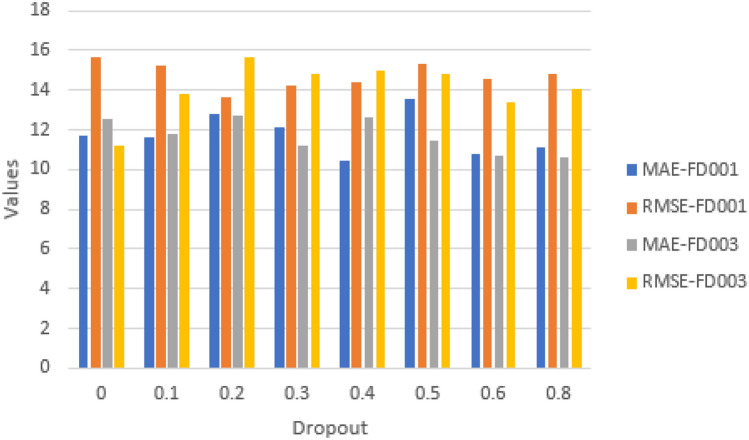
Impact of dropout on model performance on FD001 and FD003 (MAE and RMSE).

**Fig. 24:**
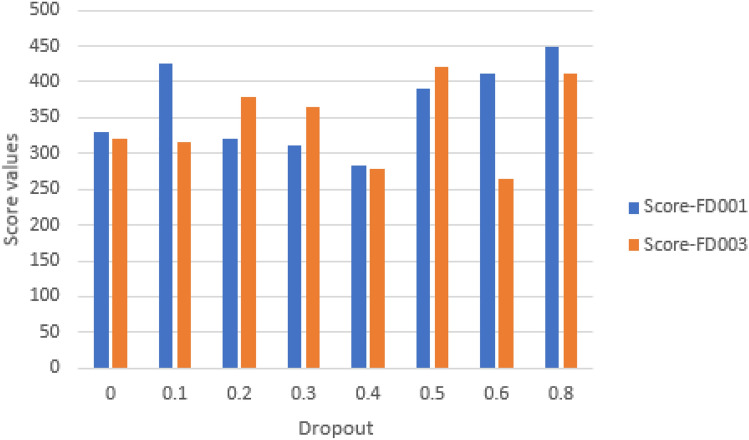
Impact of dropout on model performance on FD001 and FD003 (Scoring Function).

It is a key hyperparameter that prevents overfitting and enhances model performance. In experiments we included dropout values of 0, 0.1, 0.2, 0.3, 0.4, 0.5, 0.6 and 0.8. Figures 23 and 24 show that a dropout of 0.4 (FD001) and 0.6 (FD003) results in the least RMSE and Score for the model.

#### Comparison with different methods

The proposed method is compared with existing state of the art methods including CNN^18^, MODBNE^19^, Deep LSTM^20^, GRU LSTM^21^, RNN^23^, bi-RNN-autoencoder^24^, Attention-LSTM^25^, Chain-graph^26^, Deep quantile regression^29^, PCA-LSTM^30^, CNN-BGRU-SA^3^, VAE-RNN^32^, ABGRU^33^, CNN-LSTM-Attention^35^ and THGNN^47^ . RMSE and Score function are used to evaluate the performance of different methods. The comparison results of different methods are shown in Table 8. The proposed model results for the sub-datasets FD001 and FD003 are smaller than other methods. ABGRU^33^ has results smaller than the proposed model for FD001 and FD003 except score for FD003. CNN-BGRU-SA^3^ and Deep quantile regression^29^ have smaller RMSE and score for FD001 results than the proposed model. It outperforms other methods in some cases due to the prepossessing steps done on dataset and normalize it to be with in specific range. A linear degradation model has benefit also affect on the results of the proposed model. Using a fixed sliding window let also the proposed model obtain better results. The structure of the proposed model performs well in predicting RUL on two sub-datasets according to experimental results.

**Table 8:** RMSE and score results of different methods (N/A: values not provided).

		FD001		FD003	
Methods	Year	RMSE	Score	RMSE	Score
CNN^18^	2016	18.45	1299	19.82	1600
MODBNE^19^	2016	15.04	334	12.51	422
Deep LSTM^20^	2017	16.14	338	16.18	852
GRU LSTM^21^	2018	19.64	838.99	N/A	N/A
RNN^23^	2019	14.57	N/A	14.92	N/A
bi-RNN-autoencoder^24^	2019	14.70	273.00	N/A	N/A
Attention-LSTM^25^	2020	14.53	322.44	N/A	N/A
Chain-graph^26^	2020	17.44	468.49	N/A	N/A
Deep quantile regression^29^	2023	**13.58**	**246.59**	N/A	N/A
PCA-LSTM^30^	2021	19.43	674.79	N/A	N/A
CNN-BGRU-SA^3^	2022	**13.88**	**248**	14.85	295
VAE-RNN^32^	2022	15.81	326	14.88	722
ABGRU^33^	2023	**12.83**	**221.54**	**13.23**	279.18
CNN-LSTM-Attention^35^	2024	15.977	N/A	13.907	N/A
THGNN^47^	2024	13.15	285	12.61	255
Proposed model	2024	**14.44**	**282.38**	**13.400**	**264.47**

Significant values are in bold.

## Ablation study results of CAELSTM model without attention

We compared the performance of the full CAELSTM model, which includes the attention mechanism, to a CAELSTM without Attention). This enabled us to directly assess the impact of the attention mechanism to the model’s overall performance. To assess prediction accuracy, we tested both models using common performance metrics such as Root Mean Squared Error (RMSE), Mean Absolute Error (MAE) and score. The ablation investigation yielded the following results:

- CAELSTM with attention: RMSE of (FD001,FD003) equal (14.44,13.40), MAE of (FD001,FD003) equal (10.49,10.68) and score of (FD001,FD003) equal (282.38,264.47).
- CAELSTM without attention: RMSE of (FD001,FD003) equal (15.2261, 15.40358), MAE of (FD001,FD003) equal (11.12,11.19) and score of (FD001,FD003) equal (424.7946,272.76557).

**Fig. 25:**
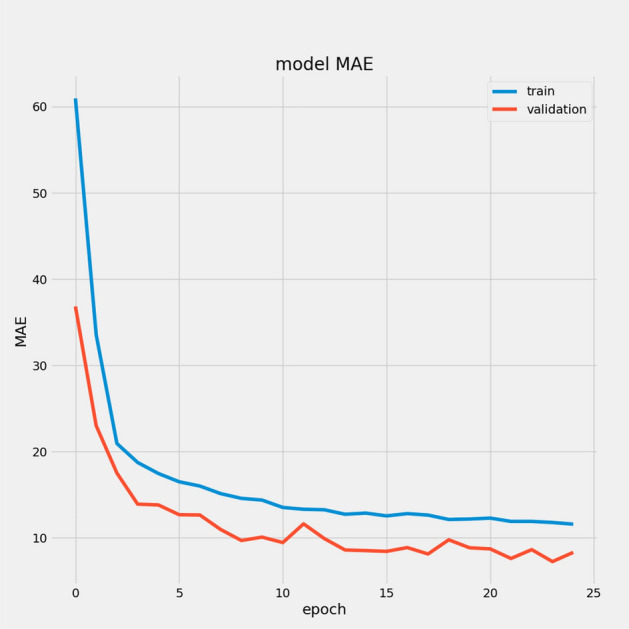
Model MAE without attention for FD001.

**Fig. 26:**
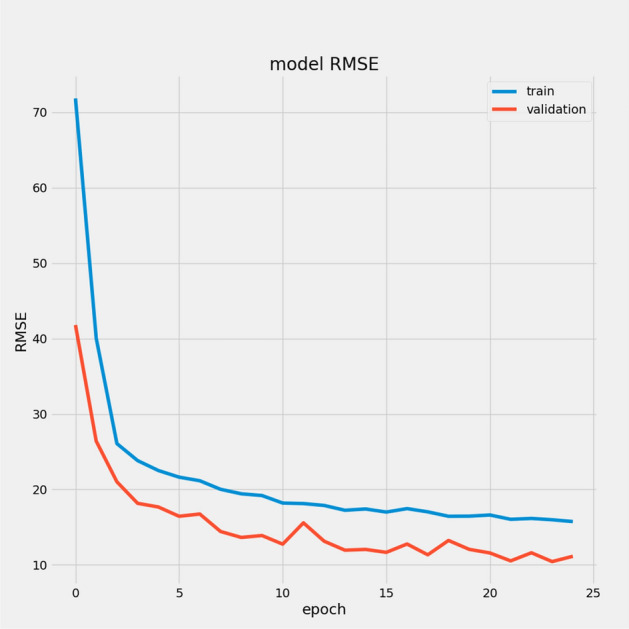
Model RMSE without attention for FD001.

**Fig. 27:**
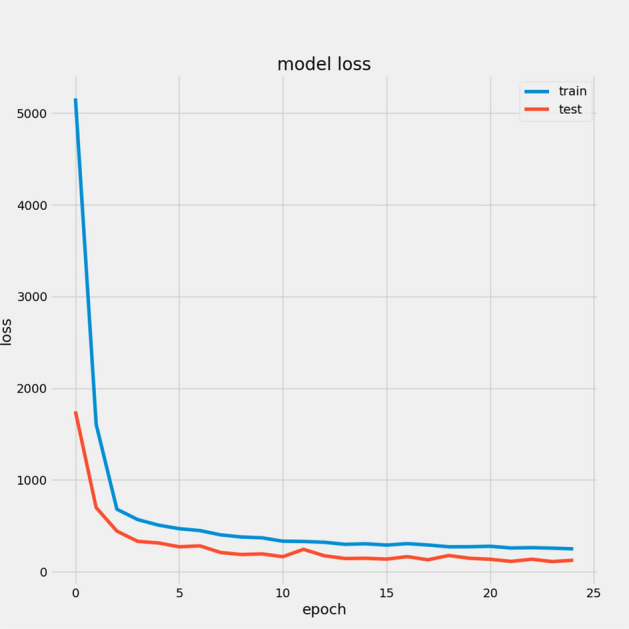
Model Loss without attention for FD001.

**Fig. 28:**
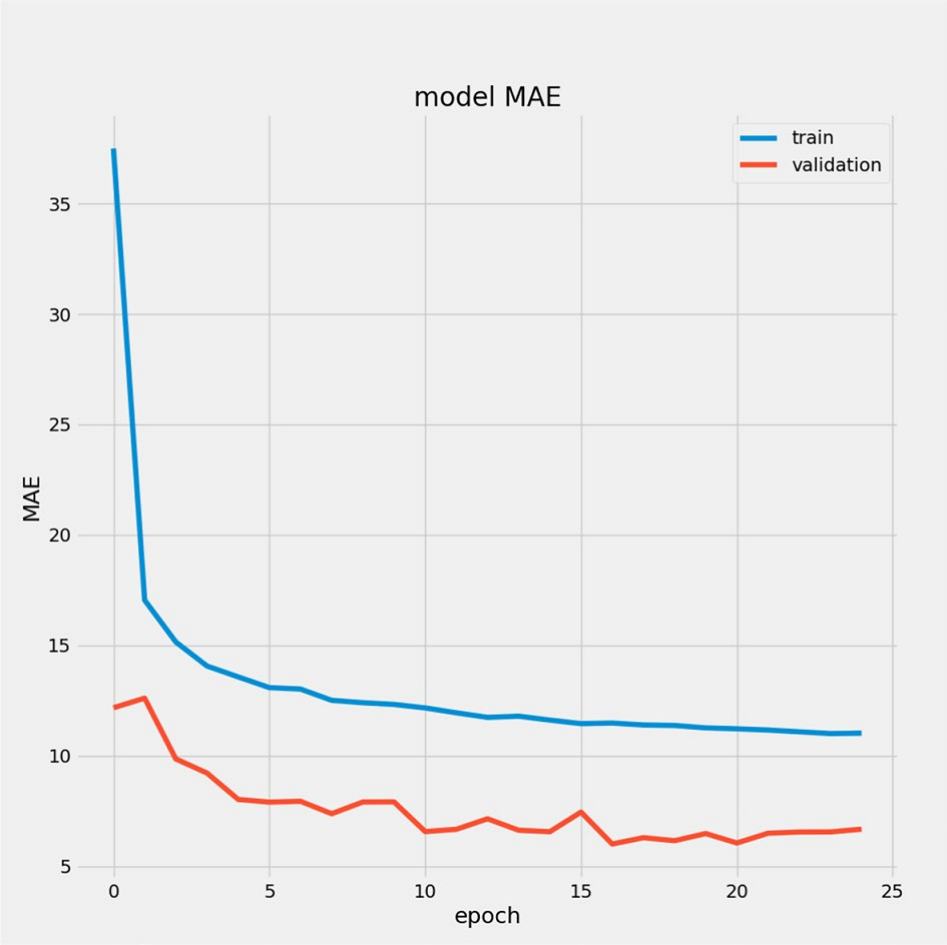
Model MAE without attention for FD003.

**Fig. 29:**
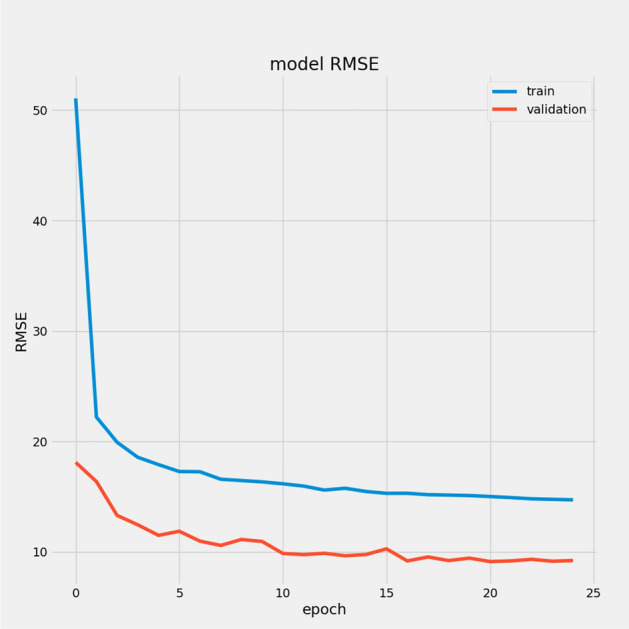
Model RMSE without attention for FD003.

**Fig. 30:**
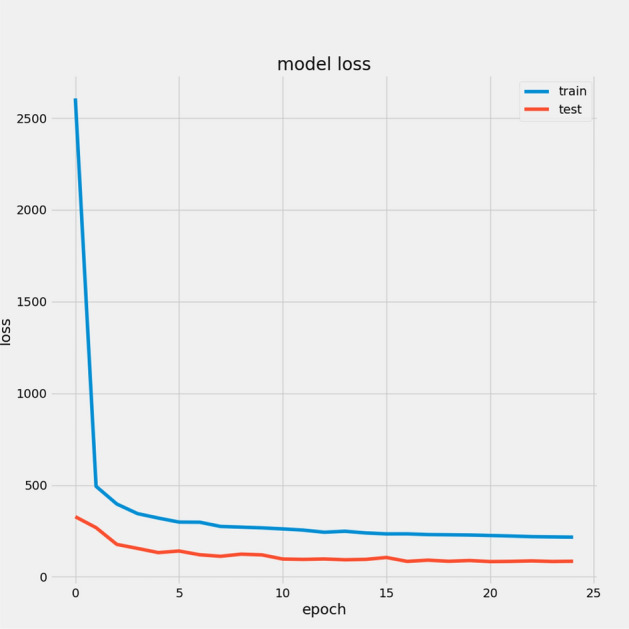
Model Loss without attention for FD003.

The results show that the CAELSTM model with attention outperformed the CAELSTM without Attention version on all criteria. The incorporation of attention lets the model to focus on the most relevant features in the input data, resulting in higher predicted accuracy. Specifically, the attention mechanism improves the model’s ability to detect major relationships in the data, which is critical for making accurate remaining usable life (RUL) forecasts. To further illustrate the impact of the attention mechanism, we have included visualizations Figs. 25, 26, 27, 28, 29 and 30 that demonstrate how the model prioritizes certain features and time steps, providing insights into the decision-making process of the model.

## Conclusion

This paper proposes a convolutional autoencoder followed by an LSTM layer with an attention mechanism and fully connected layers for predicting the remaining useful life of aero-engines. The proposed model architecture aims to learn hierarchical representations of sequential data, apply attention to focus on important parts of the sequences, and make predictions based on the processed features. The model is suitable for regression tasks where the goal is to predict a continuous target variable.

The performance of the proposed model is affected by hyperparameters such as learning rate, batch size, and dropout. Based on experiments, the results of this study established the ideal hyperparameters for the sub-datasets FD001 and FD003. We validated the proposed model using the prominent C-MAPSS dataset. The results indicate that the proposed model outperforms current approaches.

While the specifics of deployment of CAELSTM in aerospace industries may require further investigation, I believe it may be integrated into existing maintenance systems in aerospace companies. For example, it might analyse sensor data from aircraft engines and other crucial components in real time to estimate Remaining Useful Life (RUL). This predictive capability could be useful in scheduling preventative maintenance, reducing downtime and assuring safety. The proposed model has enormous potential in IoT-based situations where numerous sensors collect data from machinery and equipment. Organisations can use the model’s predictive capabilities to create apps that analyse this data in order to anticipate equipment breakdowns and optimise maintenance schedules. This would improve operational efficiency and lower the expenses associated with unexpected equipment failures.While these applications are theoretical at this point, they demonstrate the model’s potential influence on businesses that rely on predictive maintenance and real-time data processing.

To further enhance generalization of CAELSTM, future improvements could include extending the model with domain adaptation approaches to improve performance on various datasets. This is especially beneficial for industrial applications where data distributions might vary greatly. Synthetic data augmentation techniques could be used to model various degradation patterns and increase robustness. Using Bayesian deep learning or Monte Carlo Dropout could help assess prediction uncertainty and improve decision-making reliability.

Future work will focus on extending the model to the FD002 and FD004 sub-datasets, which feature more complex operating conditions and fault modes. Additionally, further optimization of the model structure and exploration of hybrid deep learning techniques will aim to enhance its generalization capabilities and adaptability to other aerospace systems. We acknowledge that Graph Neural Networks provide an effective framework for analyzing complex linkages and interactions in sensor data. Future research could focus on creating GNN-based models that exploit spatial and temporal relationships in sensor networks. By representing sensor nodes as graphs, we can capture interactions that typical sequential models may overlook. This approach could enhance the model’s ability to generalize across different sensor setups and improve its performance on tasks such as anomaly detection and predictive maintenance.

## Data Availability

The datasets used and analysed during the current study available from the corresponding author on reasonable request.
